# Supplementary material for: Recognition of Highly Branched N-Glycans of the Porcine Whipworm by the Immune System
Source: Mol Cell Proteomics. 2024 Jan 3;23(2):100711. doi: 10.1016/j.mcpro.2024.100711 (PMC10850124; doi:10.1016/j.mcpro.2024.100711)
Supplement: Supplemental Data [file mmc2.pdf]

Recognition of highly branched N-glycans of the porcine whipworm by the immune system

Barbara Eckmair[1], Chao Gao (高超)[2], Akul Y. Mehta[2], Zuzanna Dutkiewicz[1], Jorick Vanbeselaere[1],
Richard D. Cummings[2], Katharina Paschinger[1] and Iain B. H. Wilson[1,*]

[1] Institut für Biochemie, Department für Chemie, Universität für Bodenkultur,
Muthgasse 18, 1190 Wien, Austria
[2] Department of Surgery, Beth Israel Deaconess Medical Center, Harvard Medical School,
3 Blackfan Circle, Boston, MA 02115, USA

* E-Mail: iain.wilson@boku.ac.at

Supplementary Information

Further information regarding the glycomic analyses

Definition of the level of the glycan structural analysis:

The goal was the N-glycomic analysis of *Trichuris suis* adults. Thereby, individual glycan-containing HPLC fractions of glycans labelled with PA [2-aminopyridine] were subject to MALDI-TOF MS and MS/MS, in combination with a range of chemical and enzymatic treatments. In addition, glycans labelled with FMAPA [Fmoc-(3-(methoxyamino)propylamine)] were also fractionated by HPLC, analysed by MALDI-TOF MS and immobilized for use in glycan array experiments.

Search parameters and acceptance criteria

a. **Peak lists:** As stated in the methods section: typically 2000 shots were summed for MS and 4000 for MS/MS. Spectra were processed with the manufacturer's software (Bruker Flexanalysis 3.3.80) using the SNAP algorithm with a signal/noise threshold of 6 for MS (unsmoothed) and 3 for MS/MS (four-times smoothed).
b. **Search engine, database and fixed modifications:** All glycan data were manually interpreted and no search engine or database was employed; for the pyridylaminylated glycans, the fixed modification is the pyridylamine label at the reducing end ($GlcNAc_1$-PA fragments of *m/z* 300), whereas the FMAPA-labelled glycans generally show an ^{0,2}A fragment, resulting from loss of 409 Da from the parent upon MS/MS.
c. **Exclusion of known contaminants and threshold:** All glycan data were manually interpreted; only peaks with an MS/MS consistent with a pyridylaminated chitobiose core were included – the 'threshold' for inclusion was an interpretable MS/MS spectrum (at least in terms of composition).
d. **Enzyme specificity:** A description of the release methods (PNGase F followed by PNGase A) is given in the methods section. Enzymes used during the analysis (glycosyl hydrolases) are defined in the methods by species name and supplier. Citations for in-house purified recombinant enzymes are also given. As previous experience with normalizing glycosidase amounts based on units of activity towards *p*-nitrophenyl sugars reduced digestion efficiency towards native oligosaccharides, aliquots of glycans (equivalent to 5 – 50 mV in terms of fluorescence) were incubated with 0.2 µl of the various enzyme preparations (whether commercial, desalted commercial or in-house produced) overnight. These conditions result in no obvious unspecific removal of residues as defined by shifts in mass, MS/MS or retention times, although steric hindrance in some glycans leads to a requirement for longer incubation times (48 hours). Ammonium acetate buffers were used (supplemented with $CaCl_2$ where required) as suppliers' buffers interfere with MALDI-TOF MS analysis; generally one-quarter of any glycosidase digest was applied directly to the target plate prior to drying and addition of 6-aza-2-thiothymine as matrix. Hydroflouric acid treatment (3 µl of 48% HF added to the dried glycan) was 24 or 48 hours on ice in the cold room prior to drying under vacuum; expected release of α1,3-fucose and phosphodiester residues, but not of other sugars, was observed under these conditions. Incubating the N-glycans with β-galactosidases or α-*N*-acetylhexosaminidases did not affect any mass spectra.

   Fucosidases: bovine α-fucosidase (Sigma, native) removes core α1,6-fucose, but not core α1,3-fucose; almond α1,3/4-fucosidase (Prozyme, native) removes antennal α1,3-fucose from the subterminal GlcNAc of LacdiNAc, but apparently neither defucosylates chitobiose antennae nor a terminal GalNAc of a LacdiNAc motif and proved inefficient with larger structures; thus, hydrofluoric acid was more effective for the fucose removal in this study. α1,2-Fucosidases were not employed in this study.

Hexosaminidases: Jack bean β-*N*-acetylhexosaminidase (Sigma, native) is a general-purpose enzyme unspecifically removing β-linked GlcNAc and GalNAc residues, but as enzyme lots age they lose the ability to remove GalNAc from substituted LacdiNAc motifs; *Streptomyces* β1,3/4-*N*-acetylhexosaminidase (NEB, 'chitinase' recombinantly-expressed in *E. coli*) removes β1,4-linked GalNAc from LacdiNAc and β1,4-linked GlcNAc from chitobiose motifs; *C. elegans* HEX-4 is an in-house prepared enzyme (His-tag purified recombinant form expressed in *Pichia*) which demonstrably removes β1,4-linked GalNAc residues, but in this and previous studies is not observed to remove any other HexNAc; insect FDL is also prepared in-house (His-tag purified recombinant form expressed in *Pichia*) and under the stated conditions (3 hour incubation) only removes the β1,2-linked GlcNAc attached directly to α1,3-mannose in Man3-based hybrid and biantennary glycans, in keeping with its proven role in the *in vivo* generation of paucimannosidic glycans.

Mannosidases: Jack bean α-mannosidase (Sigma or Prozyme, native) removes all α-mannose residues, but steric hindrance slows its action, e.g., digestion of core α1,6-mannose is reduced if there is a 'lower' arm modification on the core α1,3-mannose; more specific α1,2-, α1,2/3- or α1,6-mannosidases were not used in this study.

e. **Isobaric/isomeric assignments:** For isobaric/isomeric species, 2D-HPLC elution, differences in MS/MS and/or digestion data were used for the assignment (as described in the text).

Glycan or glycoconjugate identification

a. **Precursor charge and mass/charge (*m/z*):** All glycans detected were singly-charged. For the positive mode, the *m/z* values for pyridylaminated glycans or FMAPA-labelled phosphorylcholine-modified glycans are for protonated forms, whereas in negative mode the ions are $[M-H]^-$ and most FMAPA-labelled glycans and all permethylated glycans are detected in the sodiated form. Depending on the glycan amount or presence of buffers in exoglycosidase preparations, the relative amounts of the H^+ and Na^+ adducts varied. Maximally two decimal places used for the *m/z* annotations consistent with the accuracy of MALDI-TOF MS; in the figures and due to space limitations, only one decimal place is presented. Previous data indicate an average +0.03 Da (+ 22 ppm) deviation between the measured and the calculated *m/z* values on the instrument used.

b. **MALDI-TOF MS settings (positive and negative modes):** Ion Source 1 and 2 were 19.00 and 16.75 kV; Lens, 9.00 kV (7.95 kV for over 3000 Da or for negative mode); Reflector 1 and 2, 21.05 and 9.65 kV; Pulsed Ion Extraction, 160 ns (120 ns in negative mode); Matrix Suppression typically up to 700 Da; Detector Gain, typically 2059 V.

c. **MALDI-TOF MS/MS settings (positive and negative modes):** Ion Source 1 and 2 were 6.00 and 5.35 kV; Lens, 2.90 kV; Reflector 1 and 2, 27.00 and 11.75 kV; Lift 1 and 2, 19.00 and 4.00 kV; Pulsed Ion Extraction, 140 ns; Detector Gain, typically 2246 V when fragmenting; Laser Power Boost typically 50%; not in CID mode; PCIS typically 0.65%.

d. **All assignments:** For the glycans present in each pool, see the HIAX and RP-amide-HPLC chromatograms annotated with structures shown according to the Standard Nomenclature for Glycans. Downwardly- and upwardly-drawn core fucose and mannose residues are respectively α1,3- and α1,6-linked (see **Figure 3**, inset, in the main text).

e. **Modifications observed:** Listed are the *m/z* values for glycans carrying a reducing terminal pyridylamine group as judged by presence of an *m/z* 300 $GlcNAc_1$-PA fragment. As the glycans are otherwise chemically unmodified, *Δm/z* of 80, 146, 162, 165 and 203 correspond to phosphate, deoxyhexose (presumed to be fucose), hexose, phosphorylcholine or *N*-acetylhexosamine. No natural methylation and no glucuronic acid residues were observed.

In the case of permethylated samples, phosphorylcholine residues were removed due to the experimental procedure.

f. **Number of assigned masses:** Glycan assignments were not just based on measured mass only, but on at least MS/MS, in most cases corroborated by digest and elution data. The linear mass spectra of higher molecular weight glycans are shown to indicate their presence but not their structure.
g. **Spectra:** Representative annotated spectra (MS and MS/MS) defining structural elements are given in various figures. In total, MS and/or MS/MS data for over 75 of the approximately 220 structures are shown in the figures, representative of all glycostructural motifs; nearly 200 mzxml files have also been uploaded to Glycopost. The overall data is based on some 2500 MS and 3500 MS MS/MS spectra.
h. **Structural assignments:** As noted in the results section, the typical oligomannosidic structures are assigned based on elution time and fragmentation pattern; it is otherwise assumed that the glycans contain a trimannosyl core consistent with typical eukaryotic N-glycan biosynthesis. The presence of an GlcNAcTIV (MGAT4) homologues in *T. suis* is compatible with the proposed tri- and tetra-antennary glycans, whose fragmentation patterns show preferential loss of the 'heaviest' antenna.
The assignments of antennal and core fucose residues are based on RP-HPLC retention time, fragmentation pattern and/or susceptibility to digestions. Other antennal modifications (phosphorylcholine and *N*-acetylgalactosamine; including anomericity of the glycosidic linkage) are defined based on digestions and fragmentation patterns with rechromatography after digestion in some cases. Based on other studies in nematodes, phosphorylcholine is assumed to be 6-linked via phosphodiesters to HexNAc or Man residues; antennal fucose and *N*-acetylgalactosamine are assumed to be α1,3-linked and β1,4-linked respectively, a combination of both only allows 6-substitution by phosphorylcholine of the GlcNAc in a fucosylated LacdiNAc motif. Core fucose is α1,6-linked, except in the case of core α1,3/α1,6-difucosylation. There is no evidence of in-source fragmentation of either neutral terminal monosaccharides (including Lewis-type fucosylation) or phosphorylcholine (as evidenced by this and previous publications).

Summary of glycosidases used in this study

PNGases	*Flavobacterium* PNGase F (recombinant, *E. coli*)<br>*Oryza sativa* PNGase A (recombinant, *Pichia*)
Fucosidases	Bovine α-fucosidase (native)<br>Almond α1,3/4-fucosidase (native)
Hexosaminidases	Jack bean β-*N*-acetylhexosaminidase (native)<br>*Streptomyces* β1,3/4-*N*-acetylhexosaminidase/chitinase (recombinant, *E. coli*)<br>*Caenorhabditis* HEX-4 β1,2-*N*-acetylgalactosaminidase (recombinant, *Pichia*)<br>Honeybee FDL β1,2-*N*-acetylglucosaminidase (recombinant, *Pichia*)
Mannosidases	Jack bean α-mannosidase (native)

Further information regarding the glycan array analyses

**1. Glycan Binding Samples**	
Description of Sample	*Fungal/plant lectins:* Concanavalin A (ConA), wheat germ agglutinin (WGA); *Aleuria aurantia* Lectin (AAL); *Ricinus communis* agglutinin 1 (RCA-I); *Sambucus nigra* agglutinin (SNA); *Lotus tetragonolobus* lectin (LTL); *Galanthus nivalis* agglutinin (GNA); *Wisteria floribunda* agglutinin (WFA); (Vector Laboratories); used at 10 µg/ml.<br>*Pathogen-associated molecular pattern receptors:* Macrophage galactose lectin (hMGL, R&D Systems); DC-SIGN (R&D Systems); Dectin-2 (Sino Biological); human C-reactive protein (biotechne); used at 1 or 5 µg/ml.<br>*Anti-glycan antibodies:* TEPC-15 (Sigma, M1421); diluted 1:200, anti-LDNF (clone F6B8); diluted 1:900, anti-FLDNF (anti-F2D2); diluted 1:50, anti-mannose AB, used at 1:400.<br>Control serum was from the pigs prior to infection; the sera of pigs infected with *T. suis* were collected at time points 21, 28, 35 and 49 days post T. suis infection; sera were diluted 1:250.
Assay protocol	The slides were incubated (all dilutions in TSMBB) with either:<br>(i) Biotinylated forms of recombinant or commercial lectins (10 µg/ml) followed by anti-biotin antibody AF488 conjugated (Invitrogen, 2 µg/ml).<br>(ii) DC-SIGN-Fc (5 µg/ml), Dectin-2-Fc (1 µg/ml) and hMGL2-Fc (5 µg/ml) followed by anti-human IgG AF 488 (Invitrogen, 2 µg/ml).<br>(iii) Human C-reactive protein (biotechne, 5µg/ml) followed by anti-CRP IgG from mouse (biotechne; diluted 1:1000 and finally anti-mouse IgG, AF 647 (Invitrogen, 2 µg/ml))<br>(iv) TEPC-15 (Sigma, C1740; 1:200, i.e., 10 µg/ml) followed by anti-mouse IgA FITC (Invitrogen, 2 µg/ml).<br>(v) Anti-LDNF antibody (clone F6B8, diluted 1:900), anti-FLDNF antibody (clone F2D2, diluted 1:50) and anti-mannose antibody (100-4G11-A) followed by anti-mouse IgG or IgM AF 647 (Invitrogen, 2 µg/ml).<br>(vi) Pig sera (1: 250) followed by mouse anti-pig IgM or anti-pig IgG (Sigma, 1:500) and finally anti-mouse IgG AF647 conjugate (Invitrogen,2 µg/ml).
Incubation and washing	Each incubation step was one hour; washing was by dipping ten times in TSMWB, then ten times in TSM and then water, followed by drying.
Sample modifications	Fluorescent antibodies (conjugated with either AlexaFluor-647/488 or FITC) were used which detect the relevant primary antibodies or which were specific for biotin in order to detect binding of biotinylated lectins.
**2. Glycan Library**	
Glycan description for defined glycans	Refer to Figure 2 for SNFG-style structures of the positive controls and selected defined natural glycans.
Glycan description for undefined glycans	N-glycans were prepared by PNGaseF/A release of glycopeptides derived by tryptic proteolysis of *T. suis* homogenates; see the experimental procedures for further details regarding isolation, FMAPA-labelling and HPLC as well as Supplementary Figures 1-4 for MALDI-TOF and HPLC profiles. A detailed analysis of pyridylaminated *T. suis* N-glycans is also presented. Normalisation was on the basis of fluorescence intensity of HPLC peaks.

Glycan modifications	Glycan pools were derivatised with FMAPA (closed ring) and the pools were HPLC purified to remove residual linker prior to re-pooling; the glycans were treated with piperidine prior to printing.
**4. Arrayer (Printer)**	
Description of Arrayer	Scienion Flexarrayer S11
Dispensing mechanism	Non-contact printing with the supplier's nozzles (Type 3) and a pulse of 45 µs, ca. 103 V.
Glycan deposition	Four replicates of 0.8 nl each, except for fractions. For FMAPA-labelled natural glycan pools, an estimated 2 nmol per spot was printed as estimated by fluorescence area of HPLC peaks in comparison to a standard.
Printing conditions	Derivatised glycans and oligosaccharides were mixed 1:1 with spotting buffer (100 mM sodium phosphate pH 8.5); printing was at room temperature; arrays were left to hybridise overnight prior to blocking (50 mM ethanolamine in 100 mM sodium borate, pH 9.0) for 1 h at RT, washing (in TSM + Tween, followed by TSM alone, and finally H_2O) and drying.
**5. Glycan Microarray**	
Array layout	8 subarrays per slide with 4 replicates per glycan sample.
Glycan identification and quality control	Glycans on the array were either pools of natural glycans or chemically/chemoenzymatically synthesized oligosaccharides. All glycans were HPLC purified and verified by MALDI-TOF MS/MS.<br>Positive controls for lectin binding were included. Spotting buffer alone was used as one of the negative controls.
**6. Detector and Data Processing**	
Scanning hardware	GenePix 4300A Scanner
Scanner settings	Multiple photomultiplier tube (PMT) gain values from 450-600 and laser power from 10-100%
Image analysis software	GenePix Pro 7
Data processing	Raw .tif image files were imported to GenePix 7.2.22; feature diameter was set to 100 µm and fluorescent intensities of spots in each sub-array were analysed. Resulting data (F525 or F635 mean values) were exported into Excel raw fluorescent values were automatically normalized and the background was automatically removed from each individual spot without subtraction; significance calculations and t-tests (parametric, unpaired, two-tailed, confidence level 95%) were performed in Excel.
**7. Glycan Microarray Data Presentation and Interpretation**	
Data presentation	Heat maps summarizing the data are shown in Figure 2. Bar charts with error bars are shown in Supplementary Figure 5. No software used for interpretation

Further information regarding the proteomic analyses

Peptide and Protein Identification

Experimental Design and Statistical Rationale

The goal was to identify the major lectin-binding band (ca. 46 kDa) observed upon Western blotting of *Trichuris suis* adult protein extract. The corresponding band from an SDS-PAGE gel was subject to a standard proteomic protocol based on trypsinization and LC-MS/MS. The experiment was performed once to identify the major protein and no statistics performed.

Protein identifications:

Accession	Description	Score Sequest HT	Coverage [%]	Nglyc Sites
A0A085N0C9	Uncharacterized protein OS=Trichuris suis OX=68888 GN=M514_10152	1733,18	83	2
A0A085N949	Uncharacterized protein OS=Trichuris suis OX=68888 GN=M513_03173	215,02	49	2
A0A085NAE4	Flavocytochrome c OS=Trichuris suis OX=68888 GN=D918_06985	172,14	70	1
A0A085NKA1	Calsequestrin OS=Trichuris suis OX=68888 GN=M514_06089	155,04	67	2
A0A085NKW0	Uncharacterized protein OS=Trichuris suis OX=68888 GN=M514_02266	130,17	13	9
A0A085LUB9	Calreticulin (Fragment) OS=Trichuris suis OX=68888 GN=M513_10576	120,38	56	1
A0A085NG03	Calreticulin OS=Trichuris suis OX=68888 GN=M513_04102	112,97	48	2

Database searching was performed using Proteome Discoverer Software 2.4.0.305 (Thermo Fisher Scientific) with the following settings:
- Protein Databases: UP_Trichuris_suis_tx68888_211110.fasta containing 24816 sequences,
  crap.fastab (www.thegpm.org/crap/)
- Enzyme: Trypsin (Full)
- Max. Missed Cleavage Sites: 2
- Precursor Mass Tolerance: 10 ppm
- Fragment Mass Tolerance: 0.02 Da
- Dynamic Modification: Oxidation / +15.995 Da (M)
- Dynamic Modification: Deamidation / +0.984 Da (N,Q)
- Dynamic Modification: Gln->pyro-Glu / -17.027 Da (Q)
- N-Terminal Modification: Acetyl / +42.011 Da (N-Terminus)
- N-Terminal Modification: Met-loss / -131.040 Da (M)
- N-Terminal Modification: Met-loss+Acetyl / -89.030 Da (M)
- Static Modification: Carbamidomethyl / +57.021 Da (C)

Decoy Database Search:
- Target FDR (Strict): 0.01
- Target FDR (Relaxed): 0.05

Peptide charge states were generally 2+ and 3+, occasionally +4, +5 and +6.
No quantification was performed.

The protein with the highest score (accession A0A085N0C9) is 90% identical to *Trichuris trichiura* poly-cysteine and histidine tailed protein (GenPept sequence ID CDW54015).

The mass spectrometry proteomics data have been deposited to the ProteomeXchange Consortium via the PRIDE partner repository (https://proteomecentral.proteomexchange.org/) with the dataset identifier PXD045512 and 10.6019/PXD045512

Assigned peptide sequences for A0A085N0C9:

Confidence	Sequence	Modifications	Theo. MH+ [Da]	XCorr (by Search Engine)	# PSMs	# Missed Cleavages
High	CCCGDYEPNEAGTECVKPPAHDCPAFGPPSEWSECLWF	6xCarbamidomethyl [C1; C2; C3; C15; C23; C35]	4872,96891	4,17	4	0
High	CCCHPYHPNEDGTACVVK	4xCarbamidomethyl [C1; C2; C3; C15]	2203,87762	5,13	7	0
High	CCCHPYHPNEDGTACVVK	4xCarbamidomethyl [C1; C2; C3; C15]; 1xDeamidated [N9]	2204,86164	4,2	4	0
High	CGYCSFR	2xCarbamidomethyl [C1; C4]	949,36548	2,25	4	0
High	CKCCCHPYHPNEDGTACVVK	5xCarbamidomethyl [C1; C3; C4; C5; C17]; 1xDeamidated [N11]	2492,98725	3,49	1	1
High	DCYPLDGQVDVCHDHGQICSLPK	3xCarbamidomethyl [C2; C12; C19]	2713,18033	4,87	4	0
High	DCYPLDGQVDVCHDHGQICSLPK	3xCarbamidomethyl [C2; C12; C19]; 1xDeamidated [Q]	2714,16435	4,87	6	0
High	DCYPLDGQVDVCHDHGQICSLPK	3xCarbamidomethyl [C2; C12; C19]; 2xDeamidated [Q8; Q17]	2715,14836	3,66	2	0
High	DGCFELR	1xCarbamidomethyl [C3]	896,39308	2,27	30	0
High	DGYAKVLMQLPYSSCK	1xCarbamidomethyl [C15]	1859,90302	4,08	2	1
High	DGYAKVLMQLPYSSCK	1xCarbamidomethyl [C15]; 1xDeamidated [Q9]	1860,88703	3,83	1	1
High	DKKDGCFELR	1xCarbamidomethyl [C6]	1267,60995	2,18	1	2
High	DNVKDCYPLDGQVDVCHDHGQICSLPK	3xCarbamidomethyl [C6; C16; C23]	3169,41358	6,61	4	1
High	DNVKDCYPLDGQVDVCHDHGQICSLPK	3xCarbamidomethyl [C6; C16; C23]; 1xDeamidated [Q/N]	3170,39759	7,75	4	1
High	DNVKDCYPLDGQVDVCHDHGQICSLPK	3xCarbamidomethyl [C6; C16; C23]; 2xDeamidated [Q12; Q21]	3171,38161	6,37	3	1
High	DWKPWTDCLWYPTQNMYTK	1xCarbamidomethyl [C8]	2533,13151	4,78	8	0
High	DWKPWTDCLWYPTQNMYTK	1xCarbamidomethyl [C8]; 1xDeamidated [Q/N]	2534,11553	4,48	18	0
High	DWKPWTDCLWYPTQNMYTK	1xCarbamidomethyl [C8]; 2xDeamidated [Q14; N15]	2535,09955	1,91	1	0
High	DWKPWTDCLWYPTQNMYTK	1xCarbamidomethyl [C8]; 1xOxidation [M16]	2549,12643	4,2	4	0
High	DWKPWTDCLWYPTQNMYTK	1xCarbamidomethyl [C8]; 1xDeamidated [N/Q]; 1xOxidation [M16]	2550,11044	3,23	7	0
High	DWKPWTDCLWYPTQNMYTK	1xCarbamidomethyl [C8]; 2xDeamidated [Q14; N15]; 1xOxidation [M16]	2551,09446	2,13	1	0
High	EKHMPHWK		1092,54075	2,76	2	1
High	EKHMPHWK	1xOxidation [M4]	1108,53566	2,61	1	1
High	EKHMPHWKR		1248,64186	2,02	1	2
High	EVGDKCKCCCHPYHPNEDGTACVVK	5xCarbamidomethyl [C6; C8; C9; C10; C22]	3020,25761	2,71	1	2
High	HMPHWK		835,40319	2,05	2	0
High	HMPHWK	1xOxidation [M2]	851,3981	2,23	1	0
High	HVEDHCHVHPDADGYRPPVATPPGIHIPEK	1xCarbamidomethyl [C6]	3377,62325	7,61	116	0
High	HVEDHCHVHPDADGYRPPVATPPGIHIPEKCGYCSFR	3xCarbamidomethyl [C6; C31; C34]	4307,97089	7,04	2	1
High	KDGCFELR	1xCarbamidomethyl [C4]	1024,48804	2,78	2	1
High	KMFESIPK		979,52811	2,43	4	1
High	KMFESIPK	1xOxidation [M2]	995,52303	2,42	3	1
High	KMFESIPKK		1107,62308	2,84	1	2
High	KMFESIPKK	1xOxidation [M2]	1123,61799	2,48	1	2
High	KSCGHDDCPTCGDICTMDK	4xCarbamidomethyl [C3; C8; C11; C15]	2256,84467	5,52	2	1
High	KSCGHDDCPTCGDICTMDK	4xCarbamidomethyl [C3; C8; C11; C15]; 1xOxidation [M17]	2272,83958	4,19	3	1
High	IAHACGLHADR	1xCarbamidomethyl [C5]	1220,5953	4,04	2	0
High	IAHACGLHADRNLTGVLGIPGEEKPPPMCGHCSFK	3xCarbamidomethyl [C5; C29; C32]; 1xDeamidated [N12]	3827,82368	4,04	1	1
High	IAHACGLHADRNLTGVLGIPGEEKPPPMCGHCSFK	3xCarbamidomethyl [C5; C29; C32]; 1xDeamidated [N12]; 1xOxidation [M28]	3843,8186	3,44	2	1
High	LKQCFTRPDTPSYVR	1xCarbamidomethyl [C4]	1867,94833	3,35	3	1
High	LKQCFTRPDTPSYVR	1xCarbamidomethyl [C4]; 1xDeamidated [Q3]	1868,93234	3,31	2	1
High	INGSCSFPR	1xCarbamidomethyl [C5]; 1xDeamidated [N2]	1038,46731	2,35	1	0
High	LPHLGCGYAFINEK	1xCarbamidomethyl [C6]	1618,80462	4,6	73	0
High	LPHLGCGYAFINEK	1xCarbamidomethyl [C6]; 1xDeamidated [N12]	1619,78864	4,17	4	0
High	LPHLGCGYAFINEKLK	1xCarbamidomethyl [C6]	1859,98365	4,51	2	1
High	LPHLGCGYAFINEKLK	1xCarbamidomethyl [C6]; 1xDeamidated [N12]	1860,96767	3,17	1	1
High	LPHLGCGYAFINEKLKQCFTRPDTPSYVR	2xCarbamidomethyl [C6; C18]; 1xDeamidated [N12]	3468,71913	5,37	2	2
High	LWDDFVAK		993,50401	2,2	21	0
High	LWDDFVAKSK		1208,631	2,4	1	1
High	MFESIPK	1xOxidation [M1]	867,42807	1,9	1	0
High	MFESIPKK		979,52811	2,02	1	1
High	MFESIPKK	1xOxidation [M1]	995,52303	2,14	1	1
High	MFESIPKKHCIEK	1xCarbamidomethyl [C10]	1646,83929	2,6	1	2
High	MFESIPKKHCIEK	1xCarbamidomethyl [C10]; 1xOxidation [M1]	1662,83421	2,61	2	2
High	QCFTRPDTPSYVR	1xCarbamidomethyl [C2]; 1xGln->pyro-Glu [N-Term]	1609,74275	4,28	26	0
High	QCFTRPDTPSYVR	1xCarbamidomethyl [C2]	1626,7693	4,5	34	0
High	QCFTRPDTPSYVR	1xCarbamidomethyl [C2]; 1xDeamidated [Q1]	1627,75332	3,09	5	0
High	SCGHDDCPTCGDICTMDK	4xCarbamidomethyl [C2; C7; C10; C14]	2128,74971	5,78	2	0
High	SCGHDDCPTCGDICTMDK	4xCarbamidomethyl [C2; C7; C10; C14]; 1xOxidation [M16]	2144,74462	3,53	1	0
High	SKEKHMPHWK		1307,66774	3,08	2	2
High	VHELHHHDHHHHHGEGHHSSSSSEDKDHHK		3550,53778	3,68	2	1
High	VHELHHHDHHHHHGEGHHSSSSSEDKDHHKDHHHK		4204,83642	5,36	3	2
High	VLMQLPYSSCK	1xCarbamidomethyl [C10]	1325,65921	2,99	31	0
High	VLMQLPYSSCK	1xCarbamidomethyl [C10]; 1xDeamidated [Q4]	1326,64322	2,6	4	0
High	VLMQLPYSSCK	1xCarbamidomethyl [C10]; 1xOxidation [M3]	1341,65412	3,3	29	0
High	VLMQLPYSSCK	1xCarbamidomethyl [C10]; 1xDeamidated [Q4]; 1xOxidation [M3]	1342,63814	2,64	1	0
High	VLMQLPYSSCKEVGDK	1xCarbamidomethyl [C10]	1853,91358	3,2	1	1
High	VLMQLPYSSCKEVGDK	1xCarbamidomethyl [C10]; 1xDeamidated [Q4]; 1xOxidation [M3]	1870,89251	2,27	1	1
High	VLQERLWDDFVAK		1618,85877	3,35	1	1

Supplementary Figure 1. MALDI-TOF MS and HPLC fractionation of FMAPA-labelled *T. suis* N-glycans. N-glycans were labelled with FMAPA and analysed by MALDI-TOF MS in positive mode prior to normal phase HPLC using an NH2 Luna column. Automatically collected half-minute fractions were again analysed by MALDI-TOF MS and pooled or refractionated on an RP-HPLC column (see ***Supplementary Figures 2-4*** for MS and example MS/MS spectra). Elution regions for the fractions **1-27** corresponding to those shown for the array data in ***Figure 2*** are indicated in addition to glycan categories. Compositions of pauci- and oligomannosidic glycans are written in green (detected as $[M+Na]^+$), simple fucosylated glycans in black (detected as $[M+Na]^+$) and PC-modified glycans in blue (detected as $[M+H]^+$). For a list of *m/z* for FMAPA-labelled glycans refer to ***Supplementary Table 1***; *m/z* differences of 146, 162, 165 and 203 correspond to Fuc, Hex, PC or HexNAc. Of the original 39 fractions, twelve with generally low lectin/antibody binding and/or significant impurities as judged by MS were excluded from the presented data.

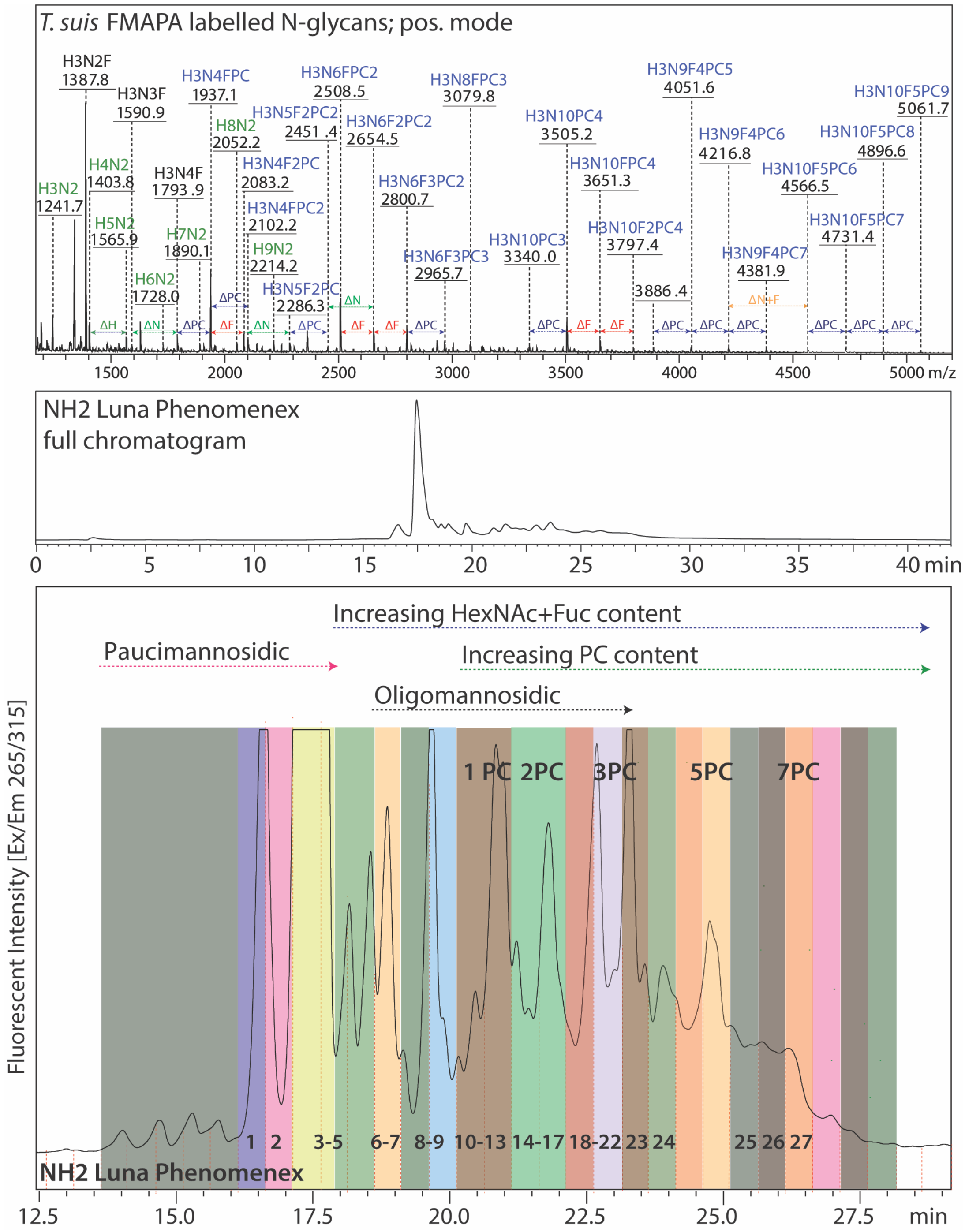

Supplementary Figure 2. MALDI-TOF MS of FMAPA-labelled *T. suis* N-glycans (fractions 1-9). NP-HPLC fractions (up to 20 min elution time; see ***Figure 1*** and ***Supplementary Figure 1***), partly refractionated by RP-HPLC, used in the glycan array experiments (***Figure 2***) were analysed by MALDI-TOF MS and MS/MS. For fractions 10-27 refer to ***Supplementary Figures 3 and 4***. While MS/MS of $[M+Na]^+$ parent ions yielded intense $^{0,2}A$ fragments (loss of 409 Da), fragmentation of $[M+H]^+$ parent ions was biased to B fragments. Major reactivities for each fraction are summarized. Glycans are depicted according to the Symbol Nomenclature for Glycans. Fractions 3-5, 6/7 and 8/9 correspond to the RP-HPLC 2D fractions for the glycans eluting at 17-20 min on the Luna NP-HPLC column. Theoretical *m/z* values are given in ***Supplementary Table 1***.

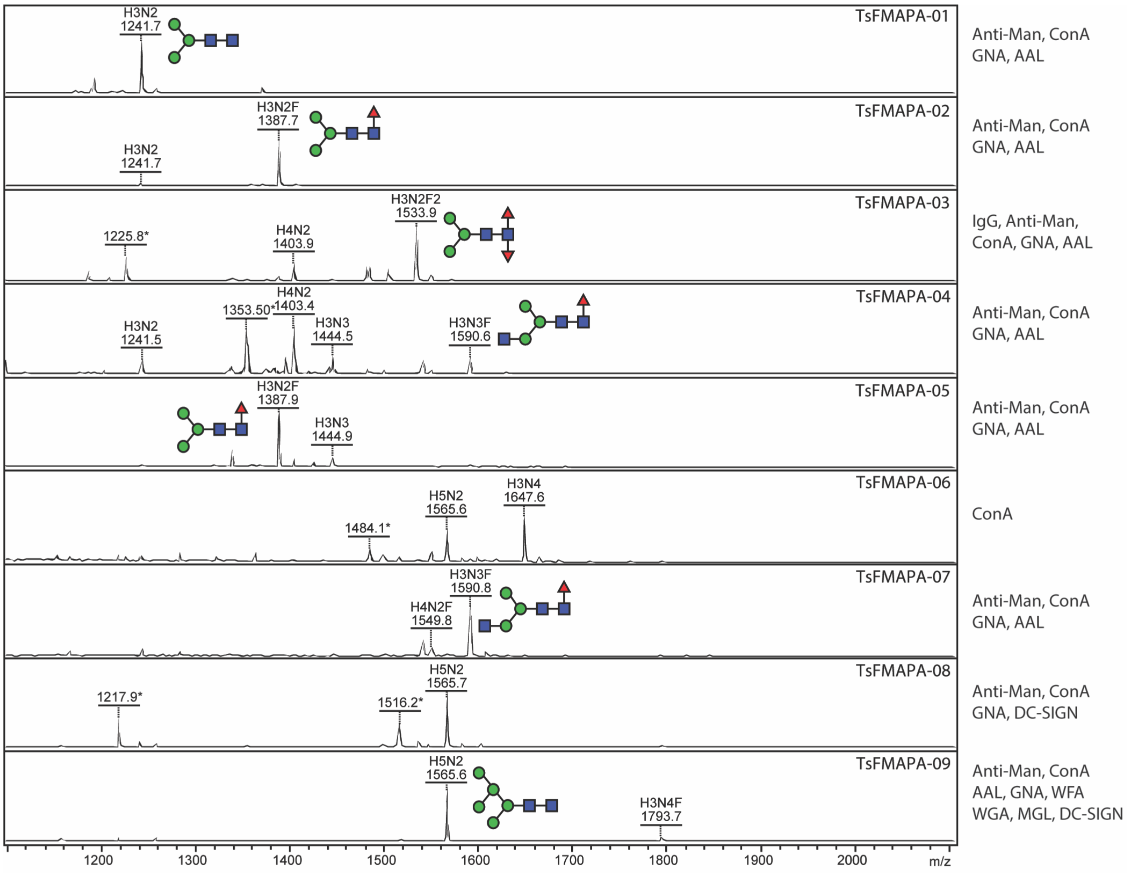

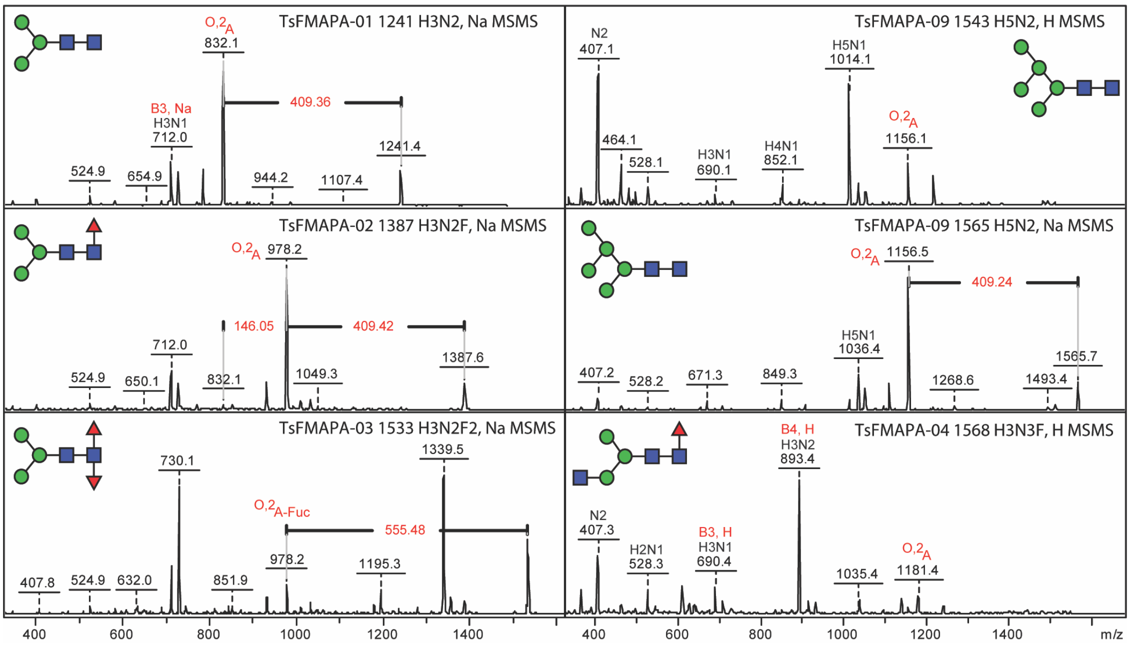

Supplementary Figure 3. MALDI-TOF MS of FMAPA-labelled *T. suis* N-glycans (fractions 10-18). NP-HPLC fractions (20-22 min elution time, see ***Figure 1*** and ***Supplementary Figure 1***, refractionated by RP-HPLC as shown) were enriched in $Man_{6-7}GlcNAc_2$ and glycans modified with 1-2 phosphorylcholine residues. Interpretation of MS/MS was aided by comparison to the corresponding PA-labelled glycans, whereby the intense PC-containing B fragment ions are informative regarding the antennal motifs (see ***Figures 4 and 5*** in the main text as well as ***Supplementary Figures 9-21***).

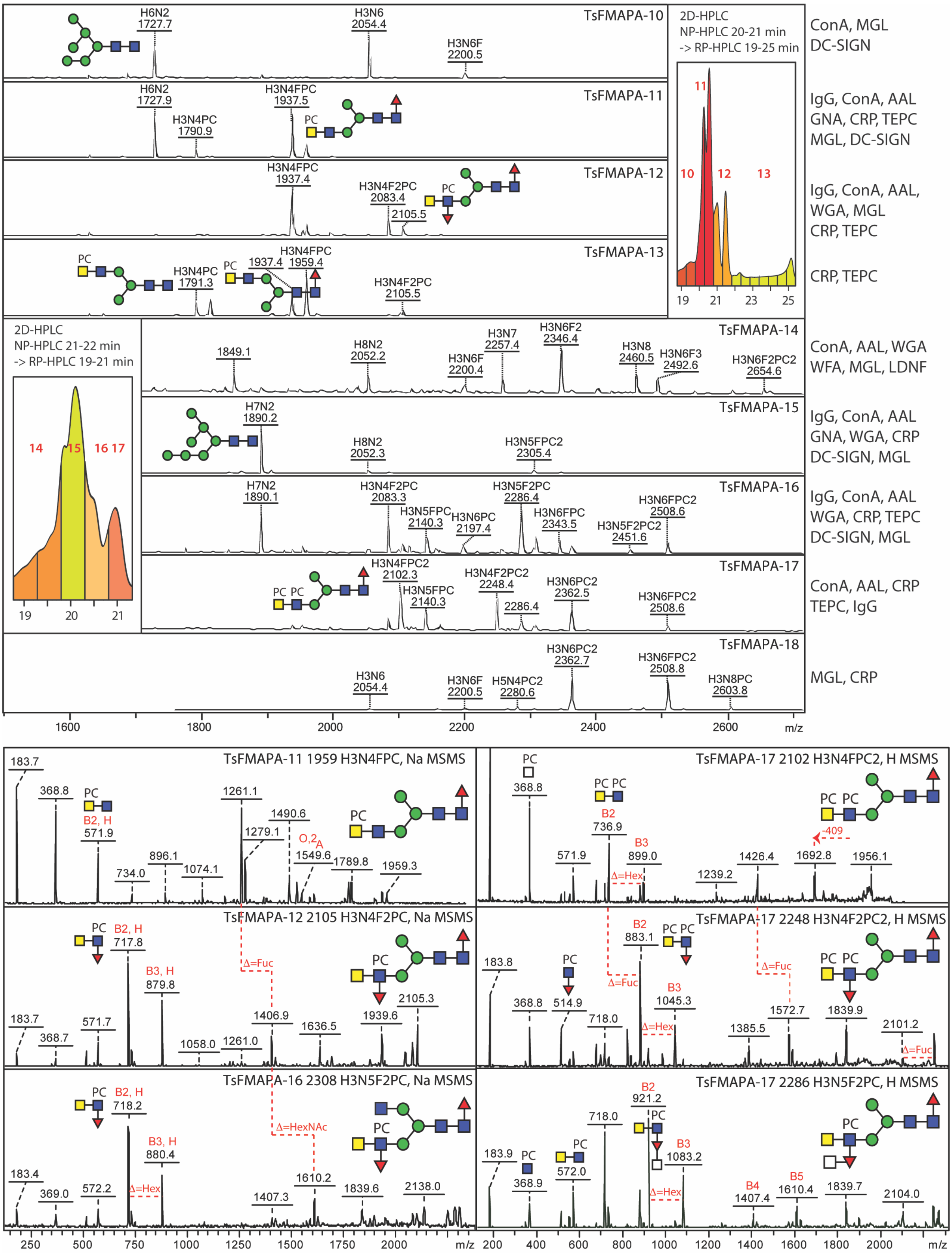

Supplementary Figure 4. MALDI-TOF MS of FMAPA-labelled *T. suis* N-glycans (fractions 19-27). NP-HPLC fractions (up to 22-26 min elution time, partly refractionated by RP-HPLC) were enriched in Man$_{8\text{-}9}$GlcNAc$_2$, Glc$_1$Man$_9$GlcNAc$_2$ and glycans modified with two or more phosphorylcholine residues. Interpretation of MS/MS was aided by comparison to similar PA-labelled glycans (see, e.g., ***Supplementary Figures 12-14***), whereby the B fragment ions and the neutral losses from parent mass are informative regarding the antennal motifs. The isomeric annotations are suggested, not definitive. The calibration of the largest glycans was performed with ACTH peptides (*m/z* 2093, 2932, 3658 and 4539). Fractions 18-20 and 21/22 correspond to the RP-HPLC 2D fractions for the glycans eluting at 22-23 min on the Luna NP-HPLC column.

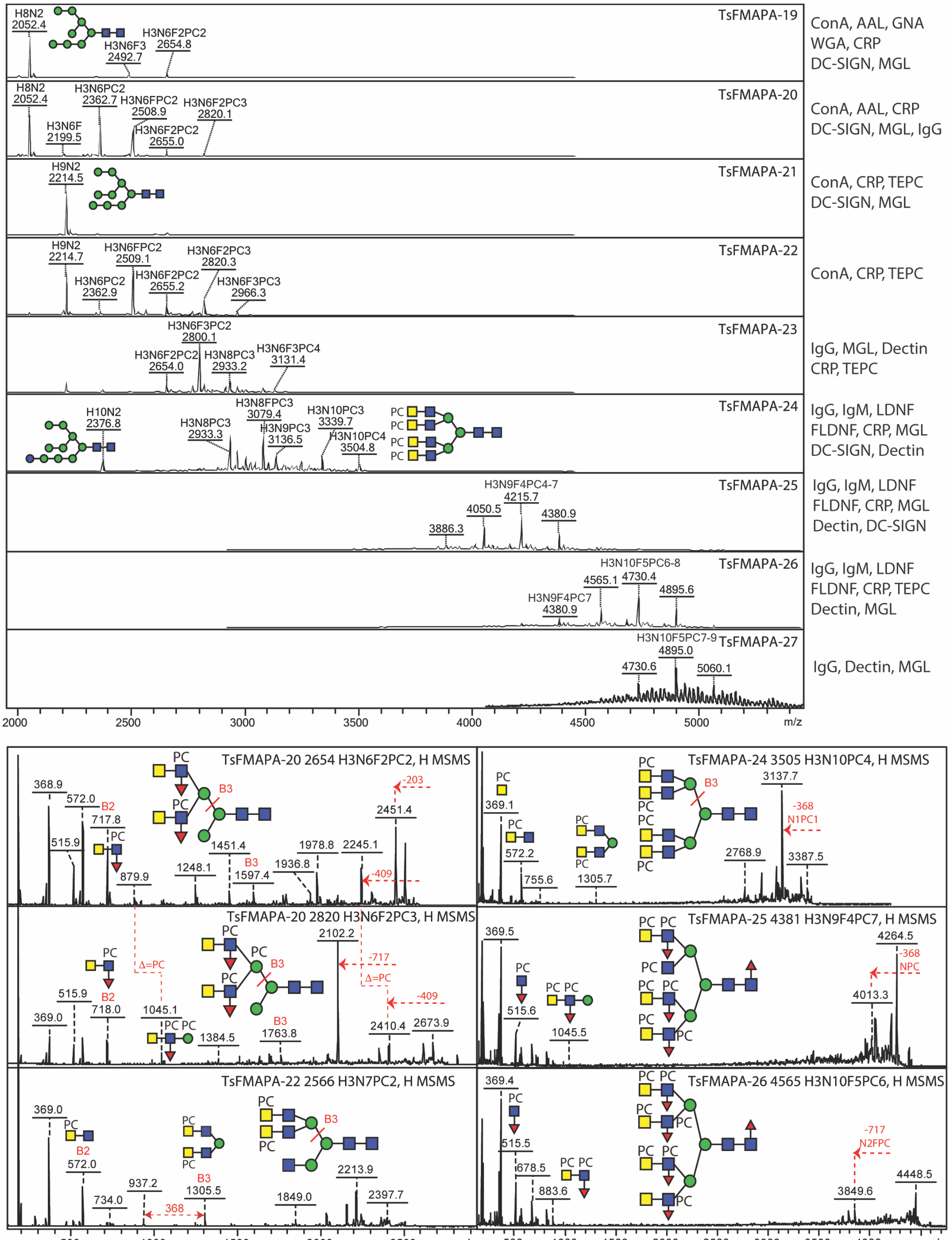

Supplementary Figure 5A. Raw glycan array data for biotinylated plant and fungal lectins; for the heat map summary and MALDI-TOF MS for the individual glycan fractions 1-27, refer to ***Figure 2*** and ***Supplementary Figures 2-4***. Standard glycans 28-32 and the buffer blank 33 are controls. Note that the maximal relative fluorescence units for SNA are 2000 as compared to 30000-70000 for the other standard lectins, reflecting a lack of sialylated glycans on the array, whereas RCA binds well to the lacto-N-tetraose control 28, but relatively poorly to any *T. suis* glycans. For details regarding the procedures, refer to the "further information regarding the glycan array analyses" section. A table with the individual data points is available as a supplementary Excel file.

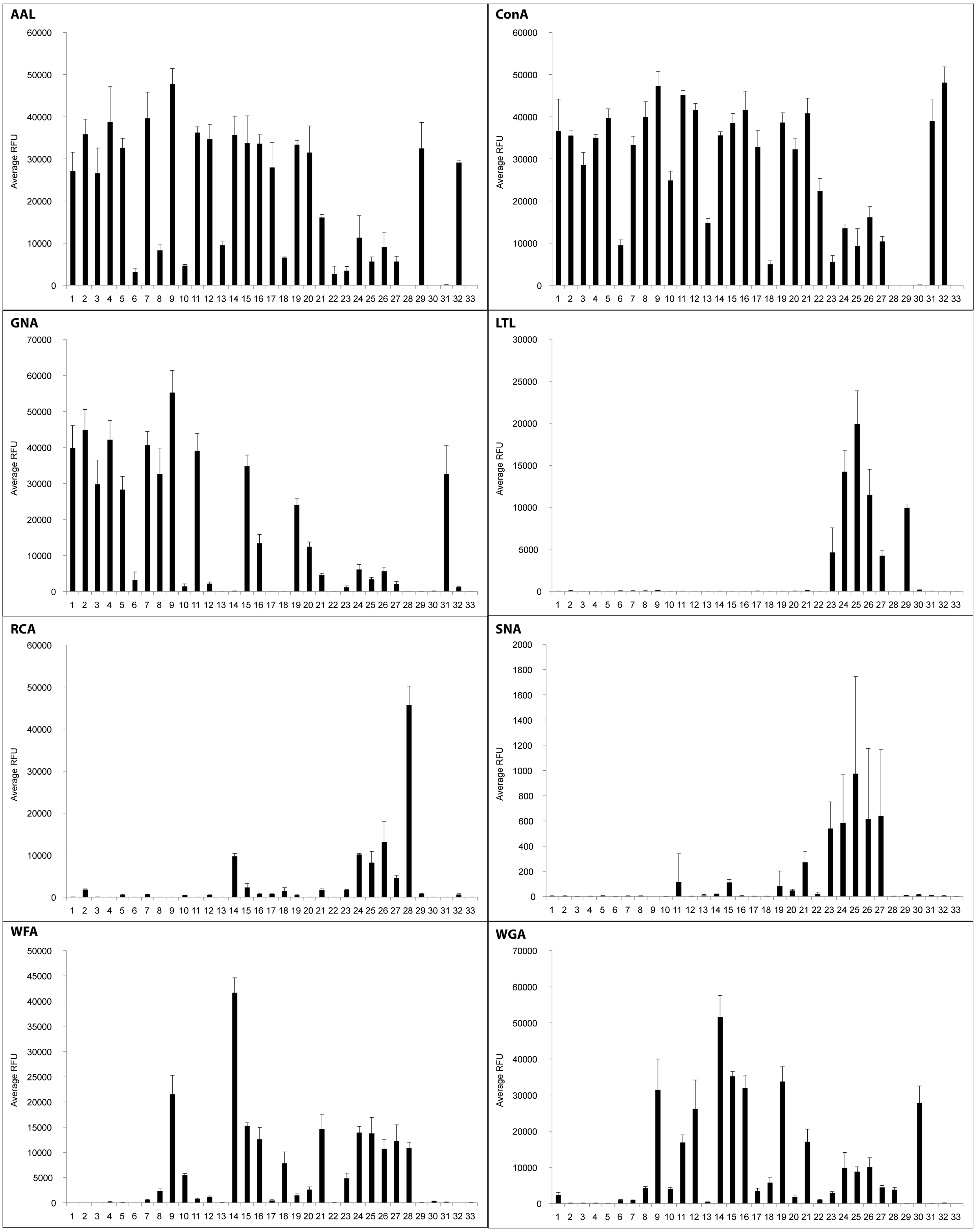

Supplementary Figure 5B. Raw glycan array data for monoclonal antibodies and mammalian innate immunity lectins. Monoclonal antibodies anti-FLDNF (F2D2), anti-LDNF (L6B8), anti-Man (100-4G11-A) and TEPC-15 (anti-phosphorylcholine) as well as mammalian lectins DC-SIGN, Dectin-2 and hMGL2 and the pentraxin C-reactive protein were employed. For the heat map summary, refer to ***Figure 2***, whereby the data for anti-FLDNF and anti-LDNF are adjusted 10- or 5-fold in the heat map comparison with anti-Man, due to the low absolute relative fluorescent units (RFU).

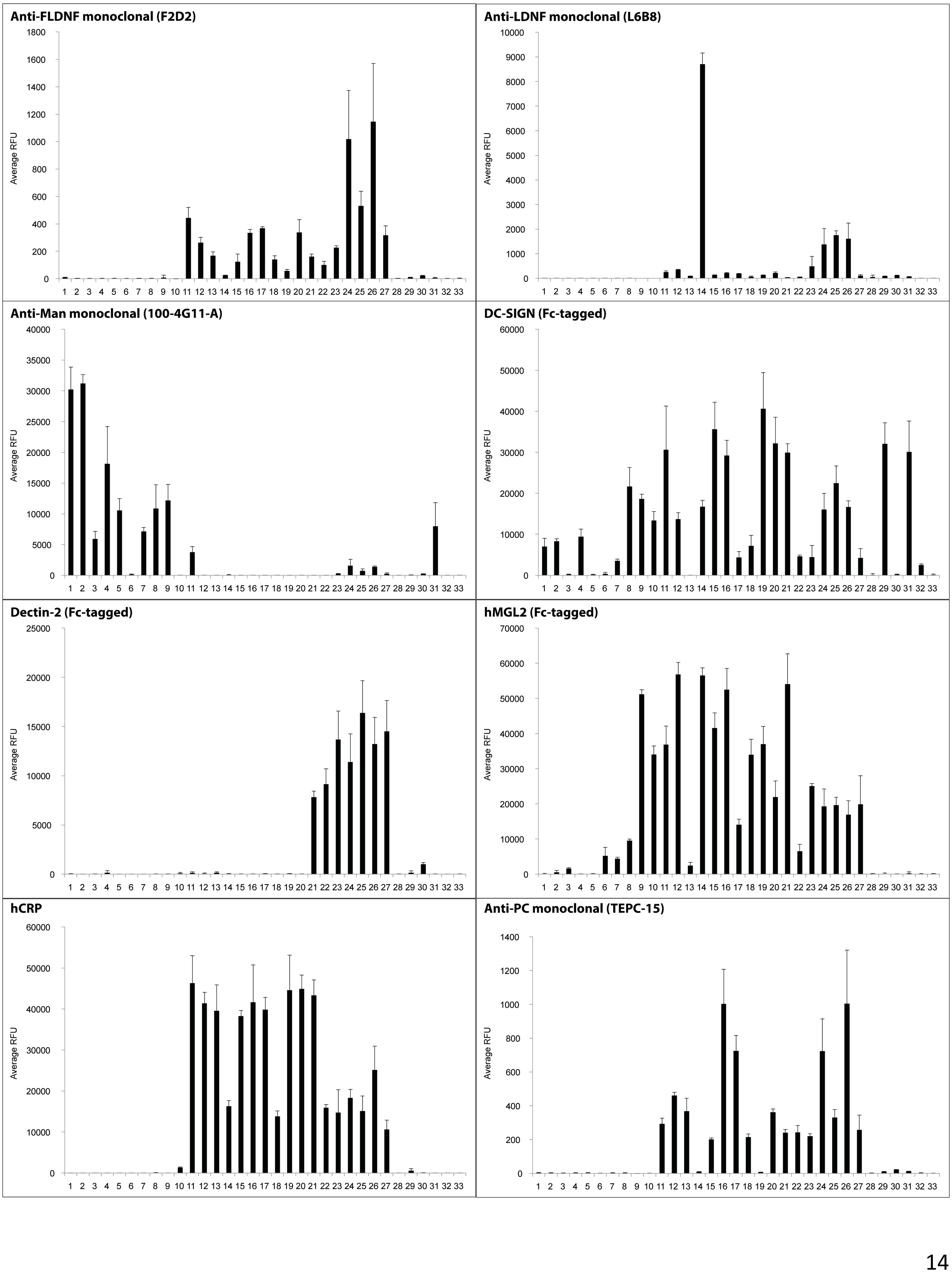

Supplementary Figure 5C. Raw glycan array data for pooled sera of uninfected and infected pigs. For the heat map summary, refer to ***Figure 2***. In absolute RFU, the highest binding of IgG and IgM is for 21-28 days post-infection and is predominantly towards the tetra-antennary N-glycans in fractions 24-26, but IgG reactivity towards a glycan with a difucosylated core (fraction 3) is observed for 28-49 days. The largest molecular weight glycans in fractions 24-26 are recognized by IgG and IgM; the IgG signal for the difucosylated glycan in fraction 3 is ca. 1000 RFU for days 28-49.

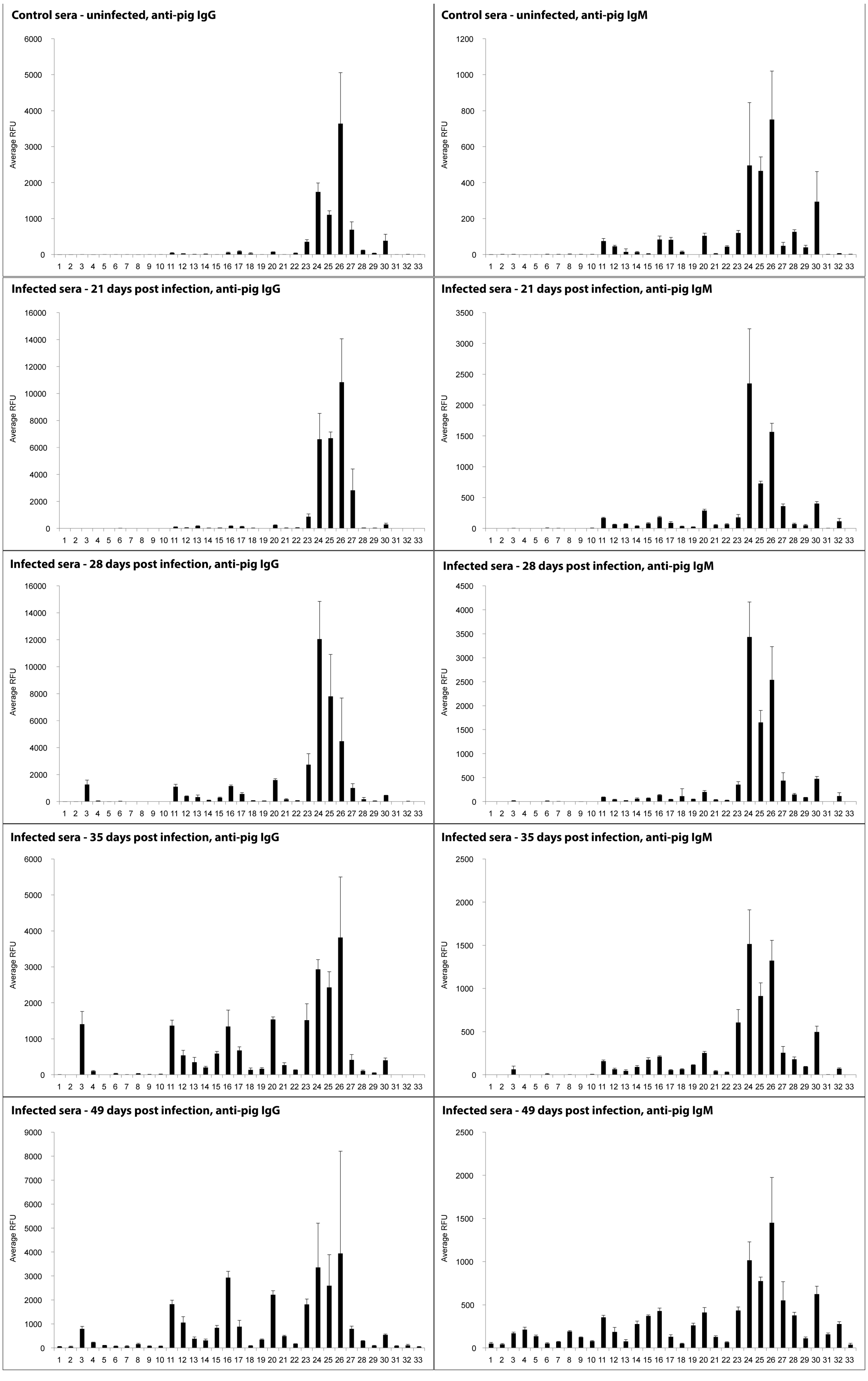

Supplementary Figure 6. Permethylation of *T. suis* N-glycans. (A and B) PNGase F and A-released N-glycans analysed by MALDI-TOF MS before and after permethylation, including the full-scale spectrum of the free N-glycans (inset) showing the dominance of the $Hex_3HexNAc_2Fuc_1$ structure. **(C)** MS/MS of a free phosphorylcholine-modified N-glycan. **(D-G)** MS/MS of four permethylated N-glycans with fucosylated LacdiNAc motifs, including a HexNAc-substituted fucose as also observed with the PA-labelled N-glycans (see ***Supplementary Figures 10 E-J and 16 G/I***). Note that phosphorylcholine is alkali labile and is not observed after permethylation. The annotated *Δm/z* of 190, 259 and 419 are interpreted as corresponding to disubstituted Hex, terminal HexNAc and a HexNAcFuc motif, while low *m/z* fragments at *m/z* 474, 701 and 946 are interpreted to respectively correspond to reducing terminal GlcNAcFuc, the fucosylated LacdiNAc and the HexNAc-substituted fucosylated LacdiNAc.

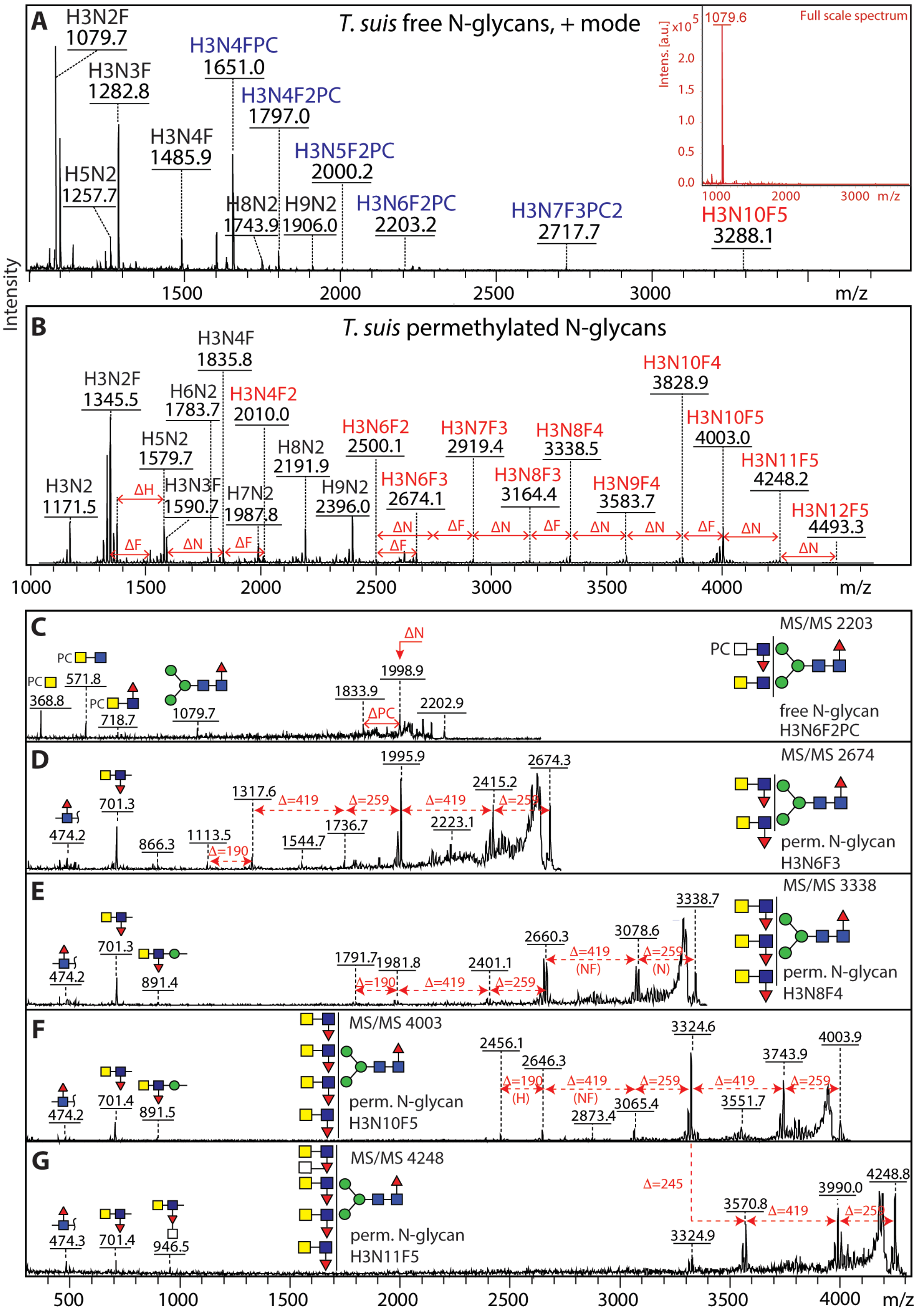

Supplementary Figure 7. 2D-HPLC of lower molecular weight *T. suis* N-glycans. Four HIAX glycan pools containing glycans of between 1100 and 2000 Da were subject to a second dimension of HPLC on an RP-amide column (fractions 9-20 from a second HIAX run, corresponding to fractions 14-30 from the first HIAX as shown in ***Figure 3***); for the simple structures, annotations are based on MS/MS and comparisons to previous elution times for the same column (e.g., the two isomers of *m/z* 1338 eluting at 8.5 and 11.5 g.u. elute similarly to those in *Dirofilaria*; Martini *et al*., 2019, *Nat. Commun*. 10, 75). However, some bi- and tri-antennary and all tetra-antennary structures elute late on RP-amide (18-20 g.u.; in grey boxes), suggesting a different isomeric structure as compared to *Dirofilaria*. The 11.5 g.u. *m/z* 1744 structure is also found in insects; defucosylation of these insect glycans co-elute with a desialylated/degalactosylated glycan derived from bovine fetuin, indicative of the lower β1,4-antenna (Kurz *et al.*, 2015, *J. Proteomics*, 126, 172). See ***Figure 4*** and ***Supplementary Figures 9 and 10*** for example MS and MS/MS data on glycans in these fractions.

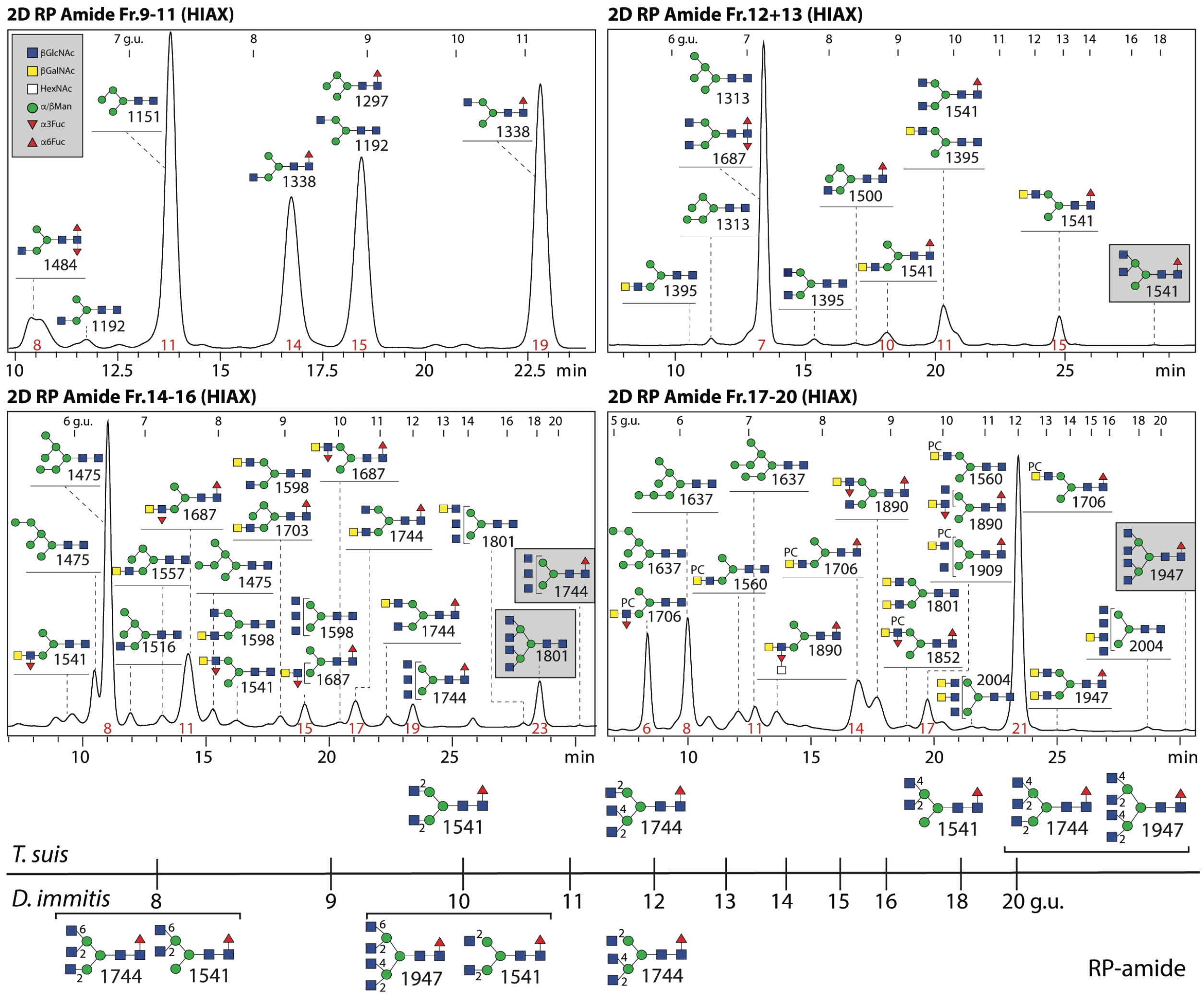

Analogous to our RP-amide data, studies on classical C18 RP-HPLC columns with pyridylaminated glycans showed that the contrasting earlier-eluting *m/z* 1744 isomers (2/2/6 and 2/4/2) eluted at 10.3 and 14.8 g.u., as compared to the typical *m/z* 1947 isomer (2/4/2/6) at 12.4 g.u.; the 'unit contribution' of lower and upper arm β1,4-GlcNAc was calculated as +2.1 and +1.3 g.u., whereas that for upper arm β1,6-GlcNAc is -2.1 g.u (Tomiya *et al*., 1988, *Anal. Biochem*. 171, 73 and Tomiya & Takahashi, 1998, *Anal. Biochem*. 264, 204). Therefore, we hypothesise that the 'upper arm' GlcNAc residues are β1,2- and β1,4-linked and that no β1,6-linked GlcNAc is found in the *T. suis* N-glycome, compatible with the presence of an MGAT4 β1,4-*N*-acetylglucosaminyltransferase gene and apparent lack of one encoding an MGAT5 β1,6-*N*-acetylglucosaminyltransferase.

Supplementary Figure 8. 2D-HPLC analysis of higher molecular weight *T. suis* N-glycans. Pyridylamino-labelled N-glycans derived from *T. suis* were fractionated by hydrophilic interaction chromatography (***Figure 3***); selected fractions were pooled and subject to RP-amide HPLC prior to MALDI-TOF MS/MS before and after chemical and enzymatic treatments (for examples, see ***Figures 4-5*** and ***Supplementary Figures 9-21***). The RP-amide column was calibrated in terms of glucose units (5-20 g.u.). Proposed structures are shown according to the Symbol Nomenclature for Glycans (see also key in grey box) and are listed in *Supplementary Table 2*; P, phosphate; PC, phosphorylcholine. The ability of RP-HPLC to resolve isomers of pyridylaminated N-glycans, as demonstrated by their different fragmentation patterns, has also been shown in previous studies on other nematode glycomes. Selected fraction numbers are shown in red. Uncertainty in terms of the antennal configurations is indicated by a bracket.

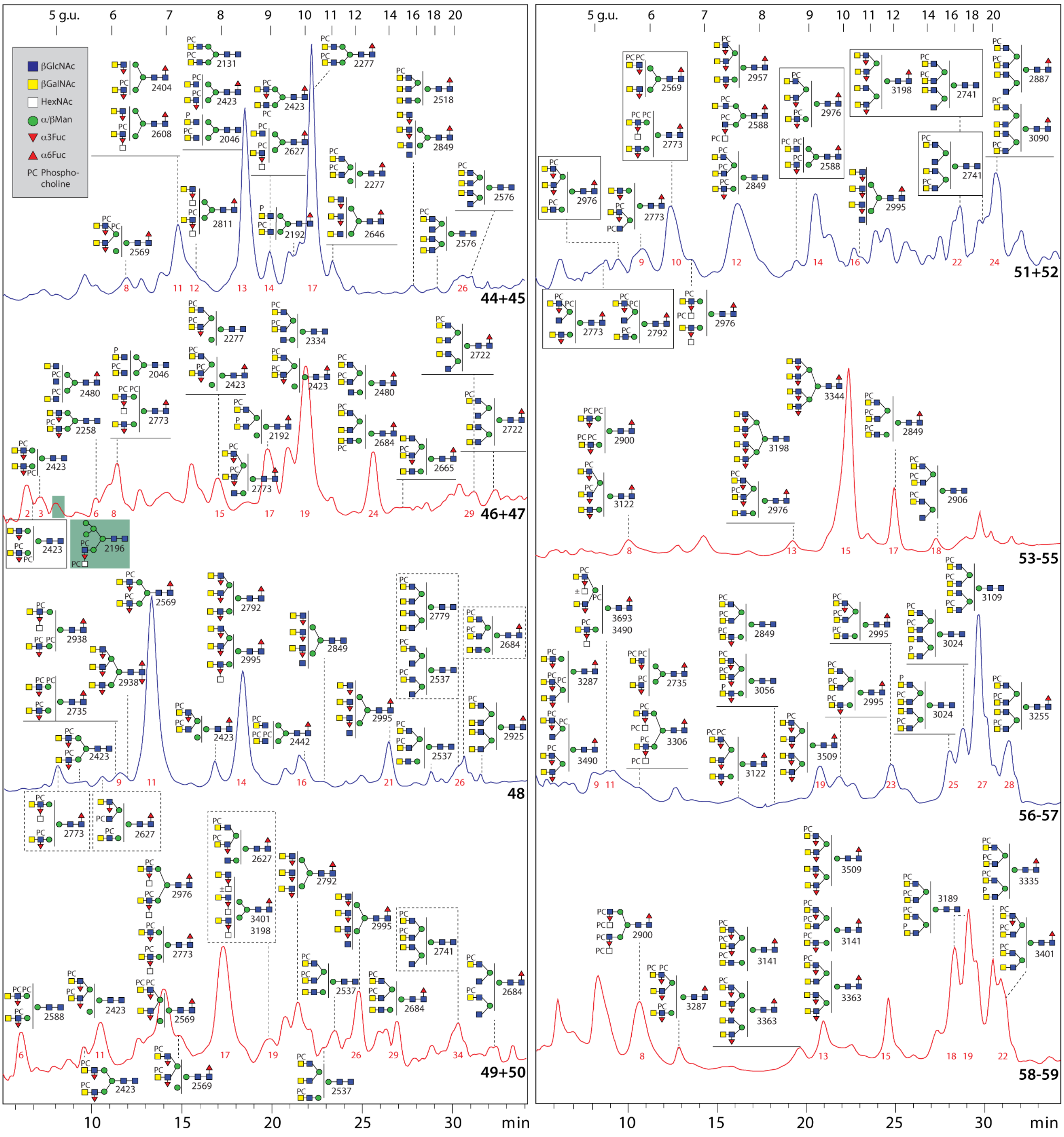

Supplementary Figure 9. MALDI-TOF MS and MS/MS of lower molecular weight *T. suis* N-glycans. (A and B) MALDI-TOF MS and MS/MS of two *m/z* 1687 isomers as well as respective enzymatic or chemical treatments to demonstrate the isomeric structures; the antennal fucose residues of the *m/z* 1687 isomers are sensitive to HF or almond α1,3/4-fucosidase; the underlying LacdiNAc motif is sensitive to chitinase or *C. elegans* GalNAc-specific HEX-4. The MS/MS comparison of the *m/z* 1687 glycans shows the difference in the ratio of the *m/z* 973 and 1135 Y-fragments depending on whether the fucosylated LacdiNAc (LDNF) motif is on the upper or lower arm. The MS/MS spectrum of the HF-sensitive *m/z* 1890 glycan is shown in ***Figure 5A***; for a comparison with the *m/z* 1687 glycan with a difucosylated core, refer to ***Figure 4A***. The two isomers have approximately the same retention time in terms of glucose units (i.e., ca. 8 and 10 g.u.) as N-glycans previously described from insects (Stanton *et al.*, 2017, *Biochim. Biophys. Acta*, 1861, 699). FAB- and GC-MS data have indicated that LacdiNAc is an underlying antennal motif in *Trichinella spiralis*, another clade I nematode (Morelle *et al.*, 2000, *Glycobiology*, 10, 941). (C) MALDI-TOF MS/MS of three *m/z* 1706 isomers showing variations in the detected B fragments; the α-mannosidase sensitivity of one *m/z* 1706 structure suggests that the free terminal mannose is the α1,3-linked residue. (D) MALDI-TOF MS/MS of two isomers each of *m/z* 1541 and 1744 with different RP-amide elution times, showing variations in the detected B and Y fragments, whereby the *m/z* 407 B fragments are indicative of a LacdiNAc motif. Refer to ***Figure 3 and Supplementary Figure 7*** for the elution positions of the relevant HIAX and 2D fractions.

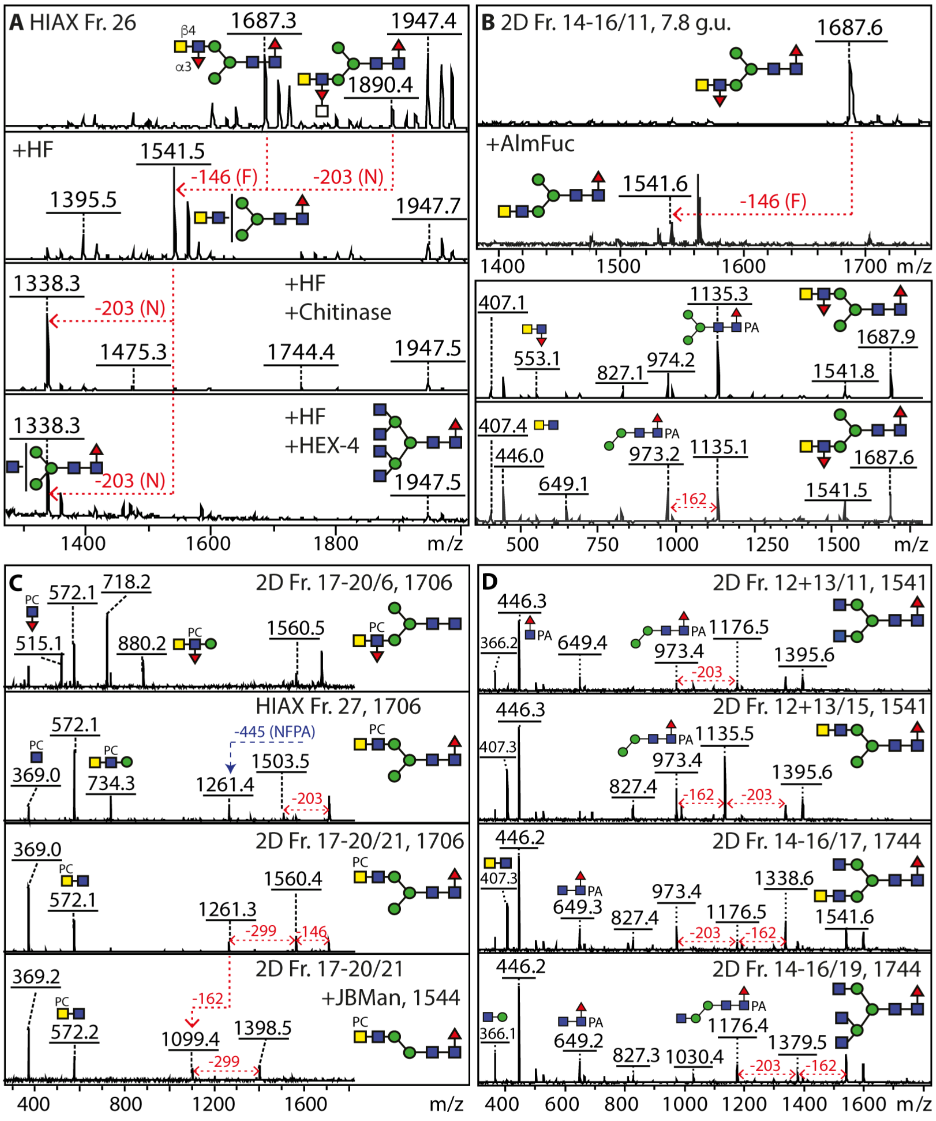

Supplementary Figure 10. Isomeric variations of LacdiNAc-modified and fucosylated N-glycans. MALDI-TOF MS/MS of selected N-glycans with LacdiNAc and fucose modifications from different 2D (HIAX followed by RP-amide) or 1D (HIAX only) fractions (see ***Figure 3*** or ***Supplementary Figures 7 and 8***). Shown are one or two isomers each of *m/z* 1801 (A), 2004 (B, C), a chitinase digestion product (D), 1890 (E, F), 2239 (G), 2792 (H), 2938 (I) and 3344 (J). Some fractions were incubated with exoglycosidases with the noted resistance or sensitivity. Antennal LacdiNAc and fucosylated LacdiNAc are characterized respectively by *m/z* 407 and 553 B2 ions, whereby the former is lost upon chitinase digestion (D); core difucosylated structures display the signature *m/z* 592 Y1 ion (I). Fractions are named with the HIAX pool number followed by the RP-amide second dimension fraction number, the *m/z* and the abbreviated composition of the form H3NxFy for $Hex_3HexNAc_{5-10}Fuc_{0-5}$. For further *m/z* 1801 and 1890 isomers refer to ***Figures 4C, 5A*** and ***Supplementary Figure 19A***; for an isobar of *m/z* 2938 refer to ***Figure 5I*** and ***Supplementary Figure 21G***.

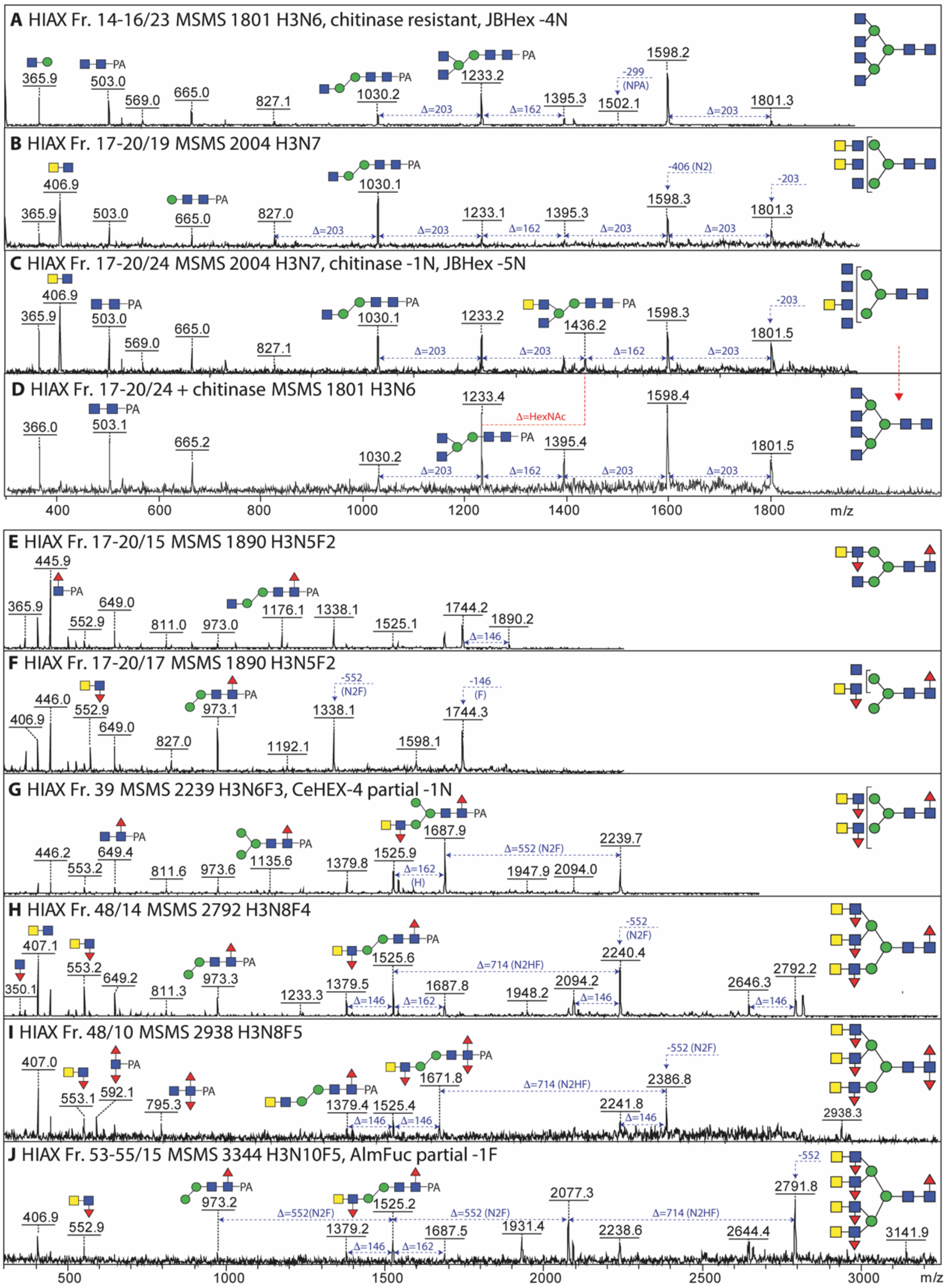

Supplementary Figure 11. Enzymatic and chemical treatments of two isomeric glycans. Two different HIAX fractions (see ***Figure 3***) containing isomeric glycans of *m/z* 1852 were subject to different combinations of HF and hexosaminidase treatments. (A-D) The *m/z* 1852 glycan in HIAX fraction 31 was sensitive to HEX-4, HF and serial HF-HEX-4 treatment; together with the MS/MS data showing an *m/z* 515 fragment ($HexNAc_1Fuc_1PC_1$) and the sensitivity of the original glycan to HEX-4, specific for β1,4-*N*-acetylgalactosamine (Paschinger et al., 2023, *J. Biol. Chem*., 299, 103053), it is concluded that the PC residue is on the same GlcNAc as the antennal fucose and not on the terminal GalNAc. (E-H) The *m/z* 1852 glycan in HIAX fraction 36 was sensitive to HF and serial HF-HEX-4 treatment, but not to HEX-4 alone; together with the MS/MS data showing the absence of *m/z* 515 fragment ($HexNAc_1Fuc_1PC_1$), it is concluded that the PC residue is on the terminal GalNAc and not on the sub-terminal fucose-substituted GlcNAc. A co-eluting phosphate-modified glycan of *m/z* 1621 displays a similar sensitivity and it is concluded that the phosphate residue substitutes the terminal GalNAc. Note that PC-modified glycans tend to ionize as $[M+H]^+$, but the digested glycans were partly detected as $[M+Na]^+$ and fragmented as $[M+H]^+$. The phosphorylcholine residue is assumed to be 6-linked considering previous data on nematode glycolipids (Friedl *et al*., 2003, *Biochem. J.* 369, 89-102), as well as considering the antennal GlcNAc residue of the *m/z* 1852 structure in the Fraction 31, which carries 2-*N*-acetyl-, 3-fucosyl and 4-*N*-acetylgalactosaminyl-substitutions, leaving only the C6 available for the phosphodiester modification. Furthermore, previous Q-TOF CAD-MS/MS and FAB-MS data have indicated that phosphorylcholine is 6-linked to HexNAc residues of perdeuteroacetylated *C. elegans* N-glycans (Haslam *et al.*, 2002, *Biochem. Soc. Symp*. 69, 117-134).

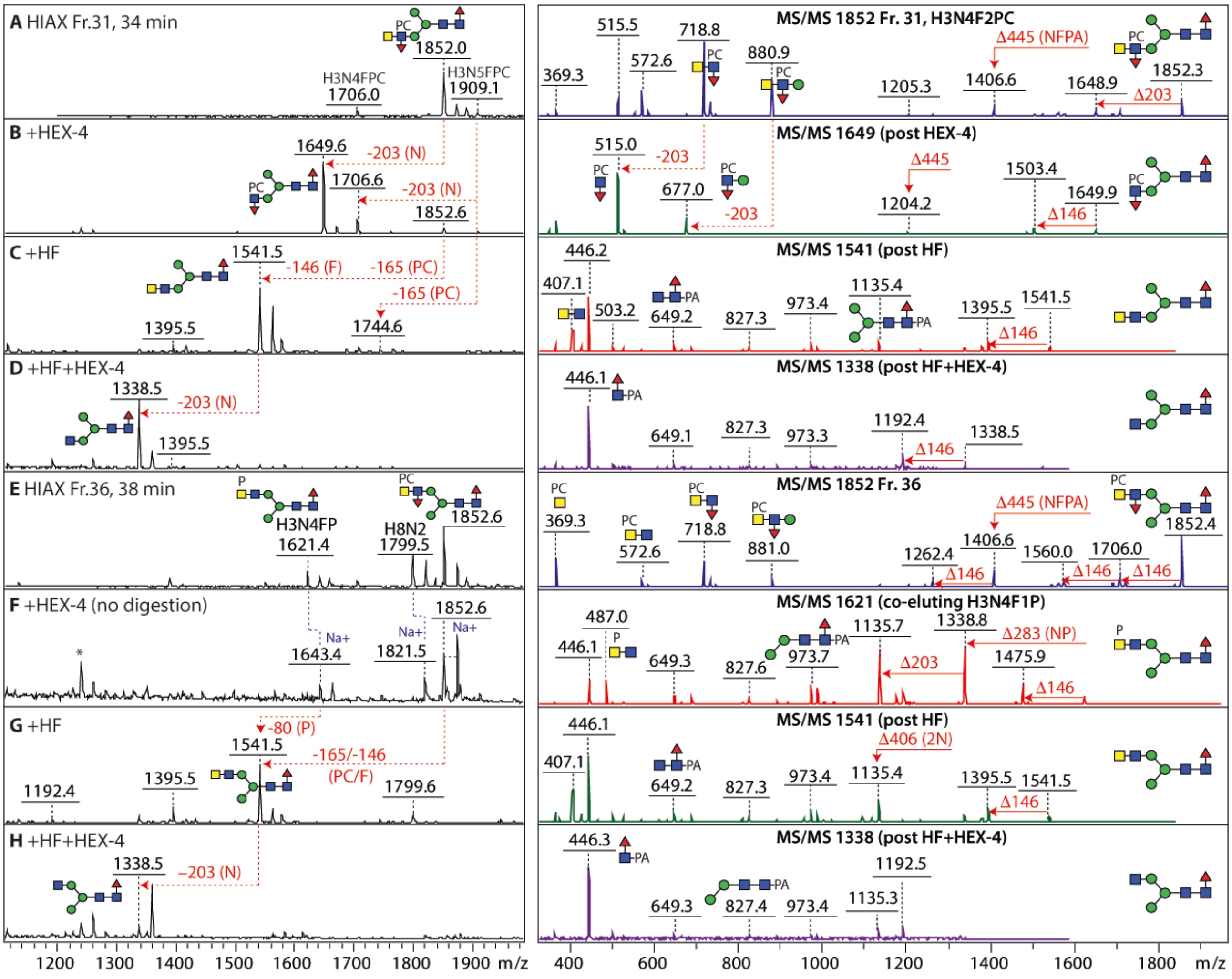

Supplementary Figure 12. Isomeric variations of phosphorylcholine-modified N-glycans. MALDI-TOF MS/MS of selected N-glycans with phosphorylcholine (PC) modifications from different 2D fractions (HIAX followed by RP-amide; see ***Figure 3*** and ***Supplementary Figure 8***). Shown are isomers of *m/z* 2423 (A-F) and 2569 (H-J), as well as one structure of *m/z* 2442 (G). The pattern of B1 and B2 ions (ratios of *m/z* 369 and 572 or *m/z* 515 and 718) as well as the loss of either 203 or 368 Da is indicative for the position of the PC on LacdiNAc and fucosylated LacdiNAc (either on the terminal GalNAc or subterminal GlcNAc) or whether both zwitterionic residues are on the same antenna (*m/z* 737 and 883). Boxes indicate the *m/z* 515 fragment distinguishing isomers with a co-substitution of GlcNAc with PC and Fuc, the *m/z* 737 and 883 fragments indicative of two PC residues and the *m/z* 1451/1597 fragments showing two antennae on the same mannose.

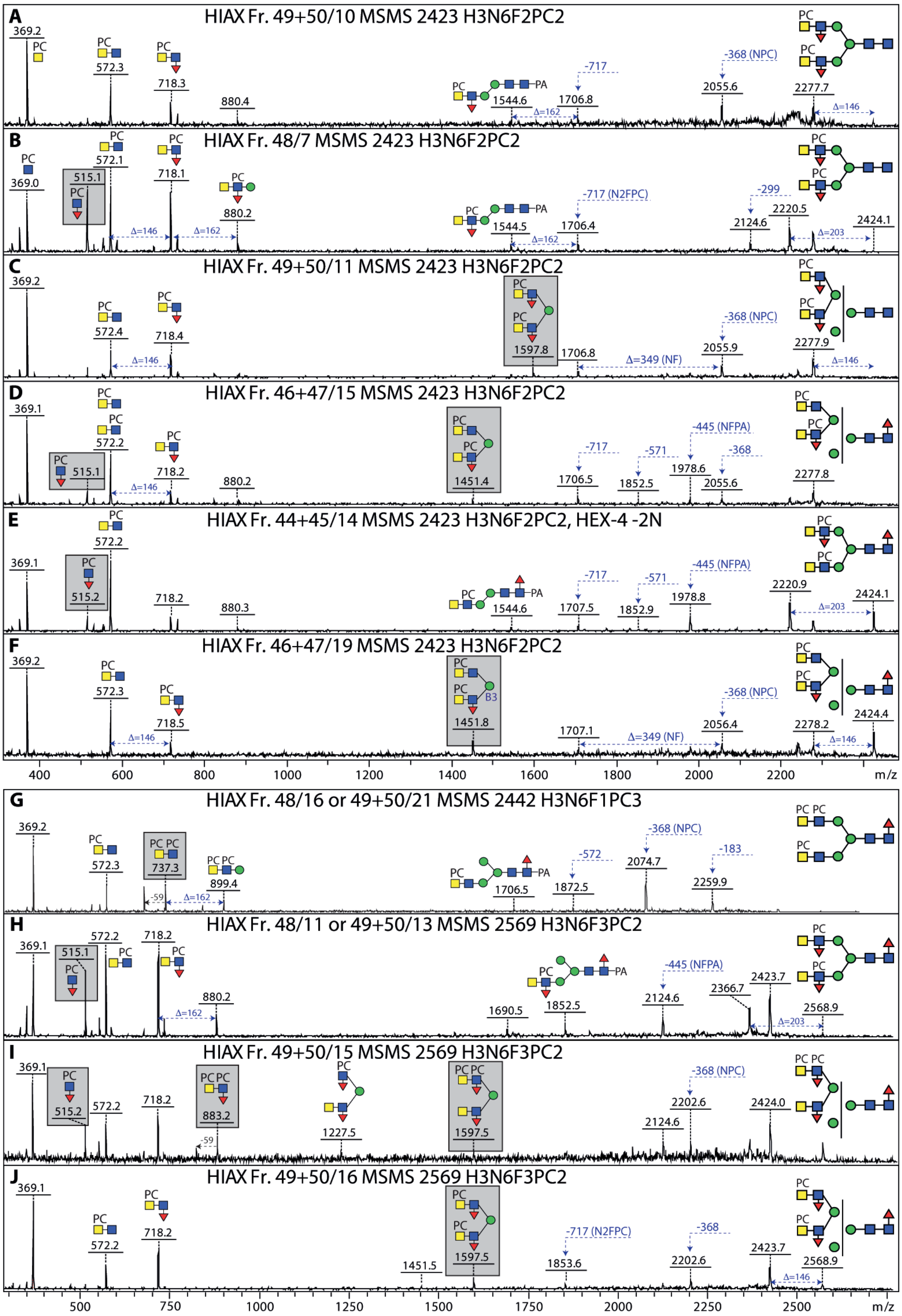

For further MS/MS of PC-modified N-glycans refer to ***Supplementary Figures 13-21***. Panels G-J are shown also in ***Figure 4 F-I*** of the main text. For further isomers of *m/z* 2423 and 2569 refer to ***Supplementary Figure 21 A-C***.

Supplementary Figure 13. 2D-HPLC and chemical/enzymatic treatment of *T. suis* N-glycans of around 3200-3400 Da. The HIAX fractions containing glycans of ca. 3200-3400 Da (***Figure 3***) were treated with HF and chitinase and then pooled prior to another round of RP-amide HPLC (see also MS and MS/MS). Subsequent jack bean hexosaminidase treatment resulted in peaks co-eluting with normal forms of $Man_3GlcNAc_2Fuc_{0\text{-}1}$, indicative that the original glycans are tetra-antennary structures. However, the intermediate late-eluting *m/z* 1947 does not correspond to the *m/z* 1947 isomer from *Dirofilaria*, suggesting that the *T. suis* tetra-antennary structure may not be one carrying two β1,2, one β1,4 and one β1,6-antennae (see note also for ***Supplementary Figure 7***), despite a 'normal' $Man_3GlcNAc_2Fuc_{0\text{-}1}$ core region, but rather possess two β1,2 and two β1,4 antennae. Note that while PC-modified glycans tend to ionize as $[M+H]^+$, the digested glycans were detected as $[M+Na]^+$ and fragmented as $[M+H]^+$.

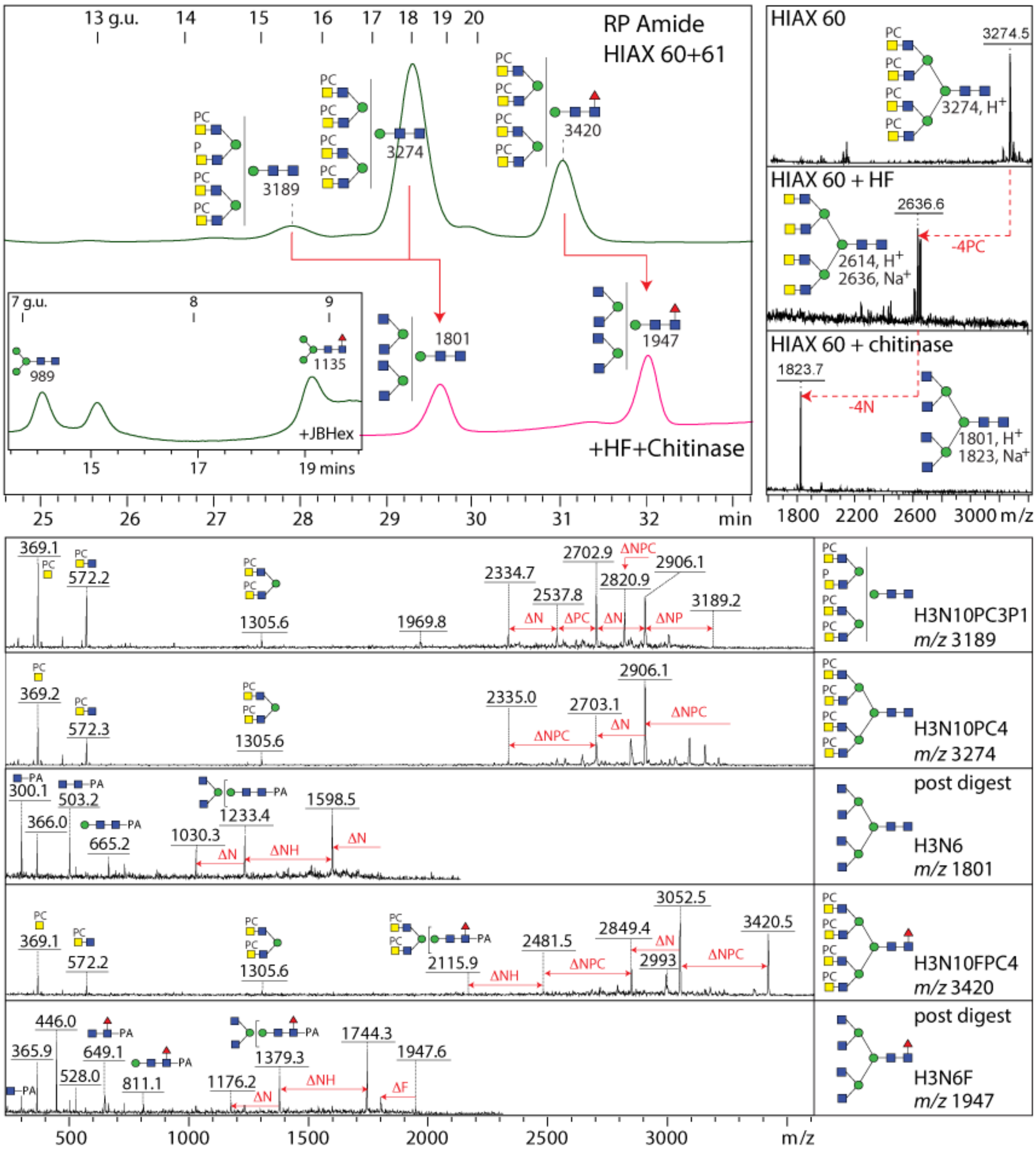

Supplementary Figure 14. HPLC and chemical treatment of *T. suis* N-glycans of 3900-4700 Da. Limited MS/MS data are available on larger N-glycans; the example for *m/z* 4499 suggests the occurrence of phosphorylcholine-modified fucosylated LacdiNAc – a tentative structure is shown. The late-eluting HIAX fractions 70-76 containing glycans of ca. 3900-4700 Da were analysed by linear MALDI-TOF MS, pooled and treated with HF prior to RP-amide HPLC, resulting in glycans of *m/z* 2354, 2557 and 2760, as defined by reflector-mode MALDI-TOF MS, eluting at 33 minutes and which were also chitinase sensitive similar to the tetra-antennary forms shown in ***Supplementary Figure 13***; note that the average masses are some 2-3 Da greater than the theoretical monoisotopic masses listed ***in Supplementary Tables 1 and 2***. Based on the observed average masses of the original glycans and the accurate masses and MS/MS of the HF-treated products, it is concluded that these glycans are maximally tetraantennary, carrying three or four antennal fucose residues and up to eight phosphorylcholine residues as compared to the tetra-antennary glycans carrying four phosphorylcholine residues in HIAX fractions 60 and 61 (see ***Supplementary Figure 13***). As the dominant B-fragment of the HF digestion product is *m/z* 407, it is concluded that the antennae are all based on LacdiNAc and not on longer chito-oligomers as found additionally in filarial species or in *C. elegans* (Martini et al., 2019, *Nat. Commun*. 10, 75; Wilson et al., 2023, *Mol. Cell. Proteomics* 22, 100505). The glycans in this region correspond to the largest glycans on the glycan array (Ts-FMAPA fractions 25, 26 and 27; see ***Supplementary Figure 4***).

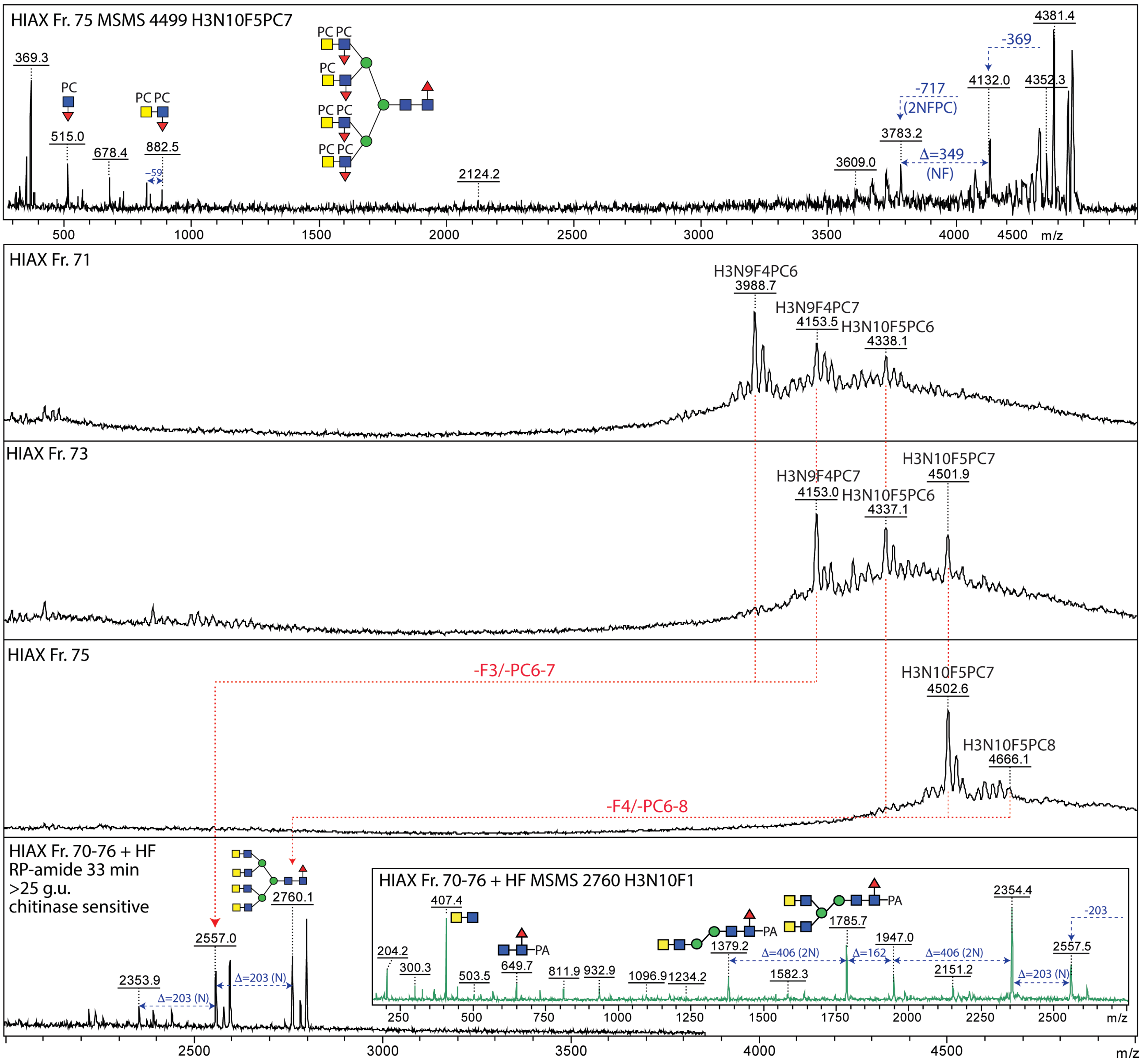

Supplementary Figure 15. Enzymatic and chemical treatments of two isomeric glycans (next page). Two different HIAX fractions containing isomeric glycans of *m/z* 2055 were subject to different combinations of HF, fucosidase and hexosaminidase treatments. (A-D) A simple biantennary form of *m/z* 2055 lost (i) a core fucose, but not the antennal fucose, upon bovine fucosidase treatment (loss of 146 Da, but no change in the B-fragments), (ii) the antennal PC-residue and the antennal fucose residue upon HF treatment, resulting in an *m/z* 1744 lacking the characteristic PC-containing B-fragments, but rather an intense B2 LacdiNAc fragment and an intense Y1 core $GlcNAc_1Fuc_1$-PA fragment; subsequent treatment with *C. elegans* GalNAc-specific HEX-4 resulted in (iii) loss of the B2 LacdiNAc fragment; note that the PC residue is indicated as modifying the GalNAc residue, due to the lack of an *m/z* 515 fragment ($HexNAc_1Fuc_1PC_1$). The co-eluting *m/z* 2093 glycan was also sensitive to serial HF and HEX-4 treatment, losing two antennal fucose and then two antennal GalNAc residues. (E-H) A hybrid form of m/z 2055 lost (i) a core fucose, but not the antennal fucose, upon bovine fucosidase treatment (loss of 146 Da, but no change in the B-fragments), (ii) was resistant to jack bean hexosaminidase unlike certain co-eluting structures with unsubstituted antennal GlcNAc residues and (ii) lost the antennal PC-residue, the antennal fucose and a HexNAc residue upon HF treatment, resulting in an *m/z* 1541 lacking the characteristic PC-containing B-fragments, but rather an intense B2 LacdiNAc fragment and an intense Y1 core $GlcNAc_1Fuc_1$-PA fragment. Note that the *m/z* 921 and 1083 B2 and B3 fragments ($Hex_{0\text{-}1}HexNAc_3Fuc_1PC_1$) lost upon HF treatment indicate the HexNAc-substitution of fucose absent from the co-eluting *m/z* 1852 glycan. Various co-eluting PC-modified and phosphorylated glycans were also sensitive to HF treatment as indicated. Only the $[M+H]^+$ ions are annotated and fragmented, but $[M+Na]^+$ and $[M+K]^+$ were also detected. For further MS/MS of structures with substituted fucose, refer to ***Supplementary Figures 16E, 19 and 20.***

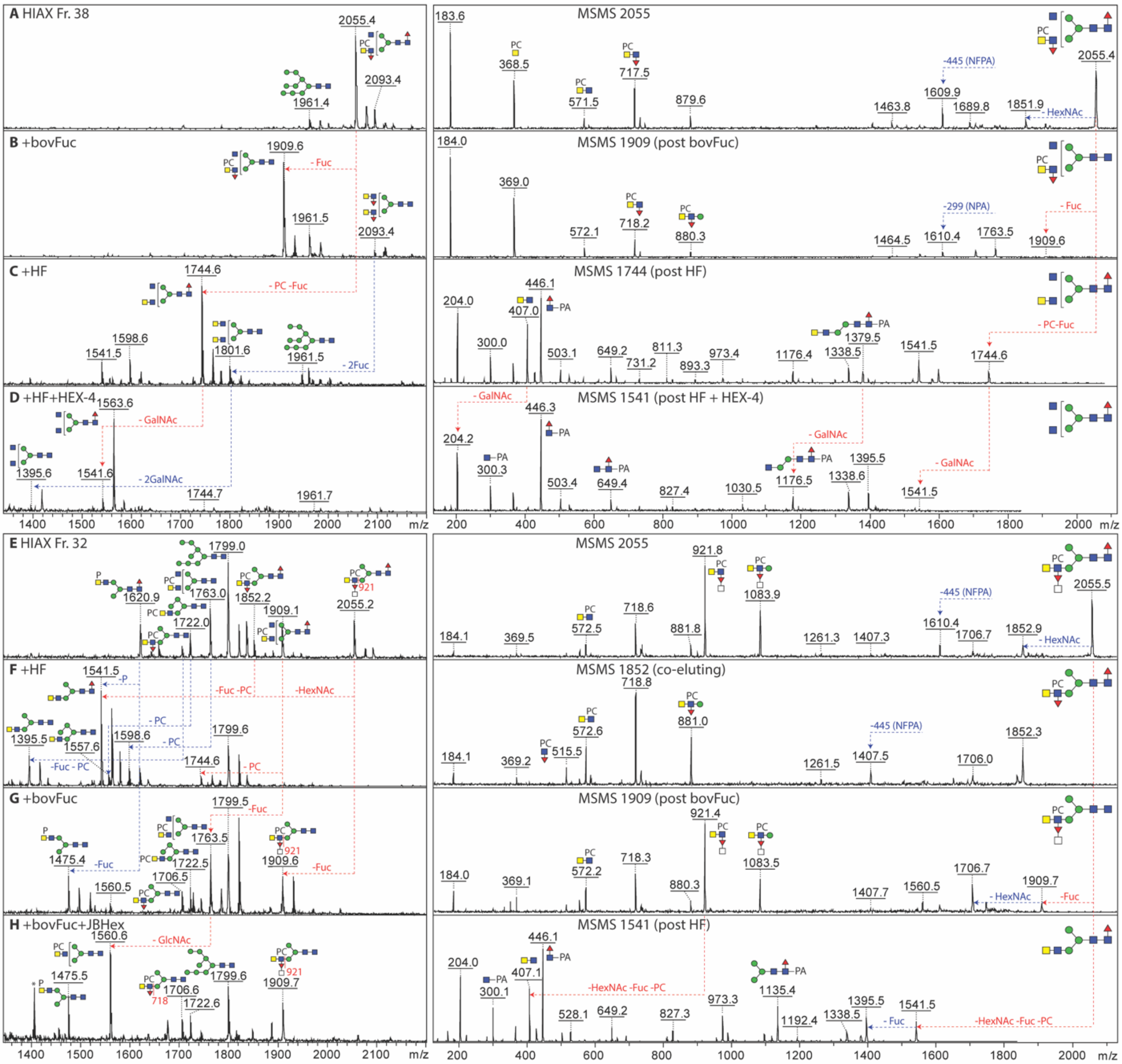
A HIAX Fr. 38
B +bovFuc
C +HF
D +HF+HEX-4
E HIAX Fr. 32
F +HF
G +bovFuc
H +bovFuc+JBHex
MSMS 2055
MSMS 1909 (post bovFuc)
MSMS 1744 (post HF)
MSMS 1541 (post HF + HEX-4)
MSMS 1852 (co-eluting)
MSMS 1541 (post HF)
- Fuc
- PC -Fuc
- 2Fuc
- GalNAc
- 2GalNAc
-445 (NFPA)
-299 (NPA)
- HexNAc
-Fuc -PC
-HexNAc -Fuc -PC
- GlcNAc
m/z

Supplementary Figure 16. Examples of isomeric and isobaric tri- and tetra-antennary glycans. (A-C) Three isomers of *m/z* 2684 in different 2D-HPLC fractions distinguished on the basis of the MALDI-TOF MS/MS B3 fragments (see grey boxes) showing the presence of different PC-containing antennae on one or both core α-mannose residues. (D-F) Three isomers of *m/z* 2773 in different 2D-HPLC fractions distinguished on the basis of the MALDI-TOF MS/MS B3 fragments (see grey boxes) showing the presence of PC-FucLacdiNAc units, with and without a HexNAc-substitution of fucose, on one or both core α-mannose residues. (G-J) Three isobaric structures of *m/z* 2995 in different 2D-HPLC fractions distinguished on the basis of the MALDI-TOF MS/MS B and Y fragments showing the presence of either fucosylated LacdiNAc, with and without a HexNAc-substitution of fucose, or PC-modified LacdiNAc; HF treatment of the isobar with the HexNAc-substitution is shown in the inset in panel H and the resulting MS/MS shows the replacement of the *m/z* 553 and 756 $HexNAc_{2-3}Fuc_1$ B2 fragments by ones solely at *m/z* 407 (LacdiNAc), comparable to data shown for PC-modified glycans in ***Figure 5 D-F*** and ***Supplementary Figures 15, 19 and 20***. The lack of a $HexNAc_3$ (*m/z* 610) fragment after HF treatment is an indication that the third HexNAc on the one antenna in panel G is indeed substituting the HF-sensitive α1,3-fucose modifying the LacdiNAc motif. All glycans fragmented as $[M+H]^+$.

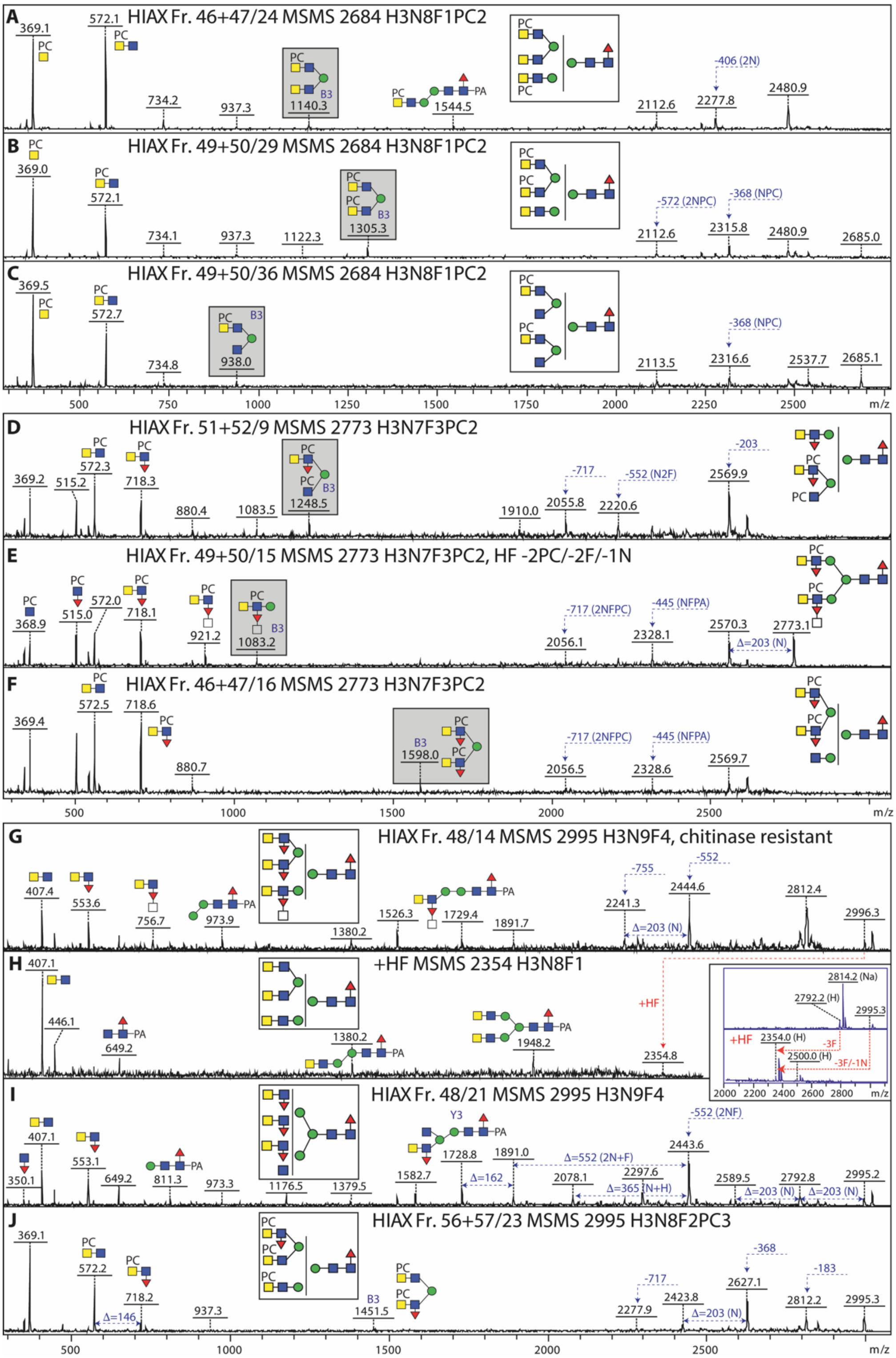

For another *m/z* 2773 isomer, with PC on Man, refer to ***Supplementary Figure 21F***.

Supplementary Figure 17. Phosphorylated and phosphorylcholine-modified N-glycans. (A-J) MALDI-TOF MS/MS of various glycans were detected which were 85 Da smaller than a phosphorylcholine-modified structure. Some of these glycans were also detected in negative ion mode (B) and showed the loss of 283 Da in positive ion mode. It was concluded that these structures are phosphorylated in one position rather than PC-modified. The patterns of B-ion fragments and losses of either 283 (GalNAcP) or 368 Da (GalNAcPC) are indicative of the antennal motifs and branching. All glycans were fragmented as $[M+H]^+$, other than the *m/z* 2190/2192 glycan also fragmented as $[M-H]^-$. B-ion fragments containing only phosphate and not phosphorylcholine are only observed here in negative mode, due to the more efficient ionization of the phosphorylcholine-containing motifs in positive mode.

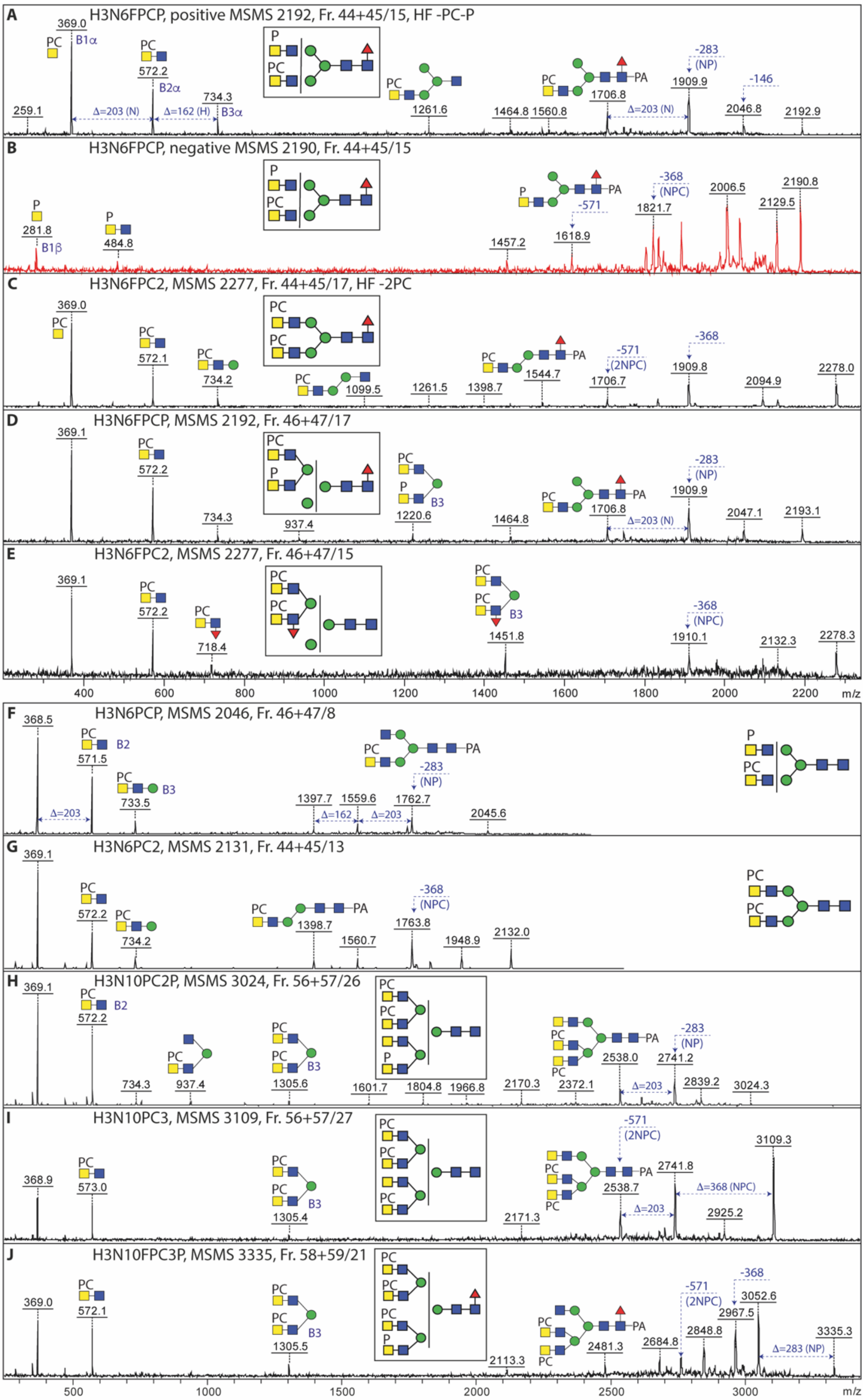

For positive mode MS/MS of a solely phosphorylated HF sensitive structure (*m/z* 1621), showing a $HexNAc_2P_1$ B ion in positive mode, see *Supplementary Figure 11F.*

MS/MS of a further *m/z* 2277 isomer is shown in *Supplementary Figure 18A*.

Supplementary Figure 18. N-glycans with phosphorylcholine and/or HexNAc-substituted fucose. (A-D) MALDI-TOF MS/MS of hybrid, tri- and tetra-antennary glycans modified with phosphorylcholine; the pattern of B fragments and losses indicate a terminal position for the PC residue, while the B3 ions show disubstitution of a core α-mannose. (E) MALDI-TOF MS/MS of a triantennary isobar of *m/z* 3401 (i.e., different composition for an almost identical mass) carrying three HexNAc-substitutions of fucosylated LacdiNAc motifs; losses of 552 and 755 Da and B ions at *m/z* 553 and 756 correspond to fucosylated LacdiNAc and its HexNAc-substituted form; for other examples, including the effect of HF treatment on this motif, see ***Figure 5*** and ***Supplementary Figures 15, 16, 19-21***. (F-I) MALDI-TOF MS/MS of two isomers each of triantennary glycans modified with phosphorylcholine and fucosylated LacdiNAc. The different pattern of smaller B fragments is indicative for a different position of one of the PC residues with the boxed B2 and B3 ions being isobar/isomer-specific.

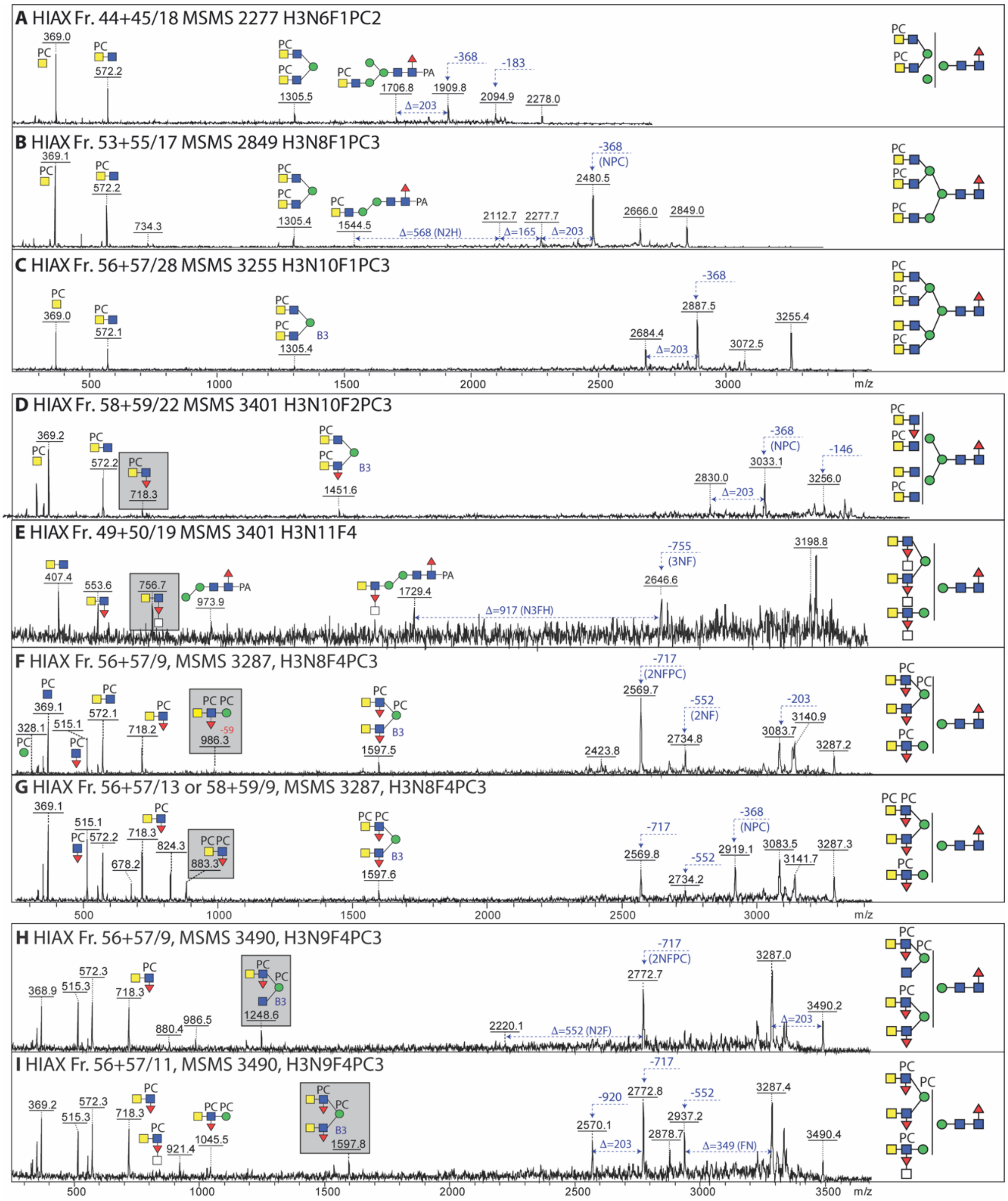

Supplementary Figure 19. N-glycans with antennal N-acetylhexosamine-substituted fucose residues. MALDI-TOF MS/MS of selected N-glycans in different 1D- or 2D-HPLC fractions (see ***Figure 3*** and ***Supplementary Figure 8***) with fucosylated LacdiNAc modifications carrying further N-acetylhexosamine and phosphorylcholine residues. A series of the simplest hybrid structures with substituted fucose residues (A-C) show differences in the numbers of phosphorylcholine residues as indicated by the B2 fragments at *m/z* 756, 921 and 1087; HF treatment of the *m/z* 1890 and 2055 glycans (see also ***Supplementary Figures 9 and 15***) resulted in loss of the FucHexNAc and PC units. The co-eluting biantennary structures (D-F) of *m/z* 2404 and 2608 were both digested down to *m/z* 1947 with HF (see inset) accompanied by loss of the B2 ions at *m/z* 718 and 921, indicative of the loss of a FucHexNAc unit from the latter. (G and H) PCHexNAc substitution of antennal fucose in the context of hybrid and biantennary glycans; for further examples, see also ***Supplementary Figures 16 E/G, 20 and 21 F/G***, while MS/MS for two further *m/z* 1890 and 2055 isomers are shown in ***Supplementary Figures 10 and 15***. Panels A-F are also shown in ***Figure 5*** of the main text.

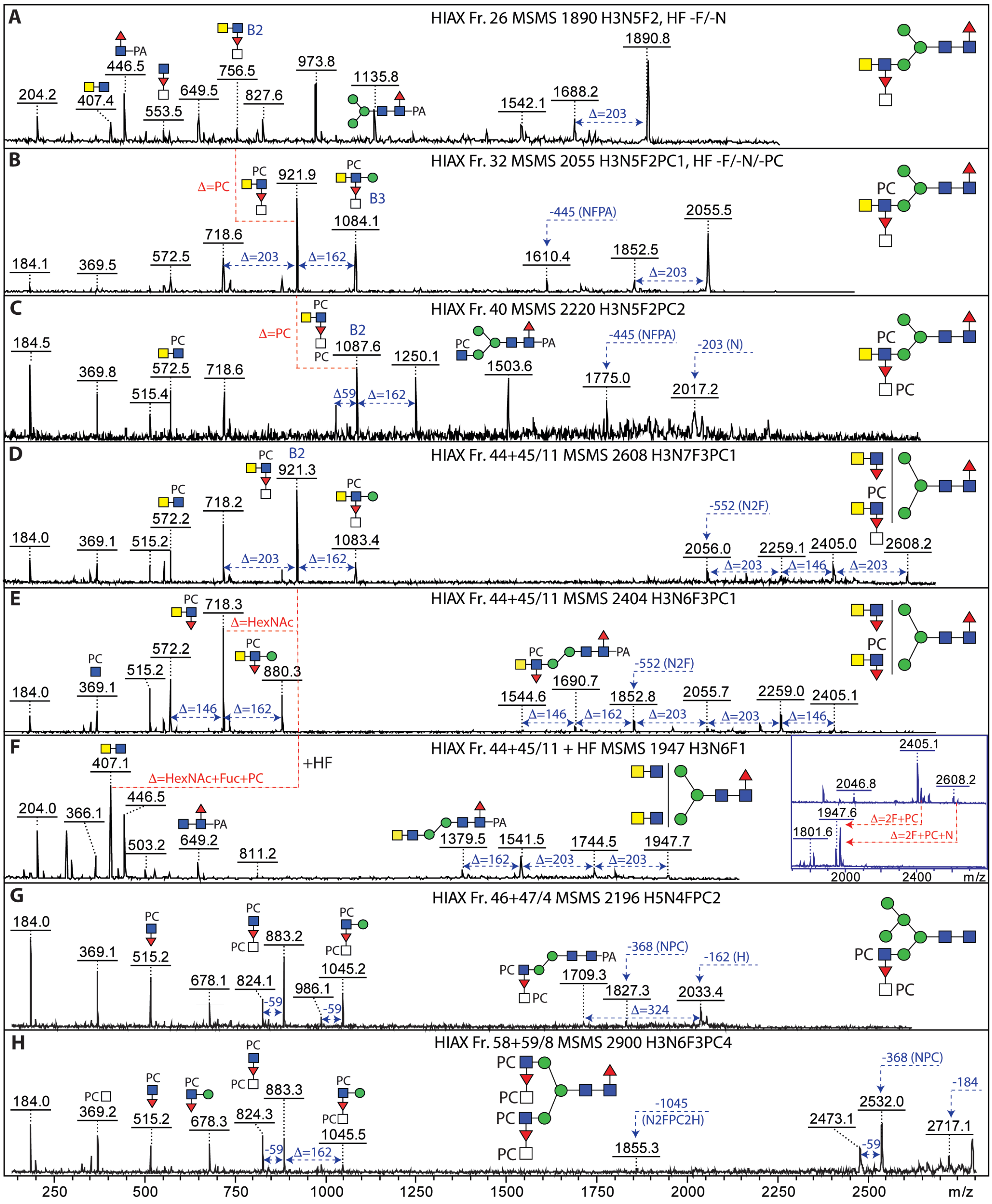

Supplementary Figure 20. Chemical treatment of biantennary N-glycans with HexNAc-substituted fucose. (A and B) The 2D-HPLC fraction containing glycans of *m/z* 2773 and 2976 was treated with hydrofluoric acid, resulting in a major product of *m/z* 1947 (*m/z* 1969 as $[M+Na]^+$). Both these isomers have either one or two HexNAc-substituted fucose residues in the context of PC-modified LacdiNAc motifs, which are replaced by an *m/z* 407 $HexNAc_2$ B-fragment after HF treatment. As indicated in ***Supplementary Figure 15 E-H***, lower molecular weight glycans with the *m/z* 921 fragment were also HF treated, losing a HexNAcFuc motif as well as a PC moiety, replaced by a B fragment at *m/z* 407; as shown in ***Supplementary Figure 9***, the similar motif lacking the phosphorylcholine was also HF sensitive (loss of HexNAcFuc) and a GalNAc residue is lost upon subsequent treatment with HEX-4, indicative that the HexNAcFuc motif is a modification of a LacdiNAc antenna. For other examples of HexNAc-substituted fucose residues, refer to ***Figures 5*** and ***Supplementary Figures 16, 18, 19 and 21***. The linkage and the nature of the HexNAc residue are unknown, but this modification has not been detected in any nematode to date.

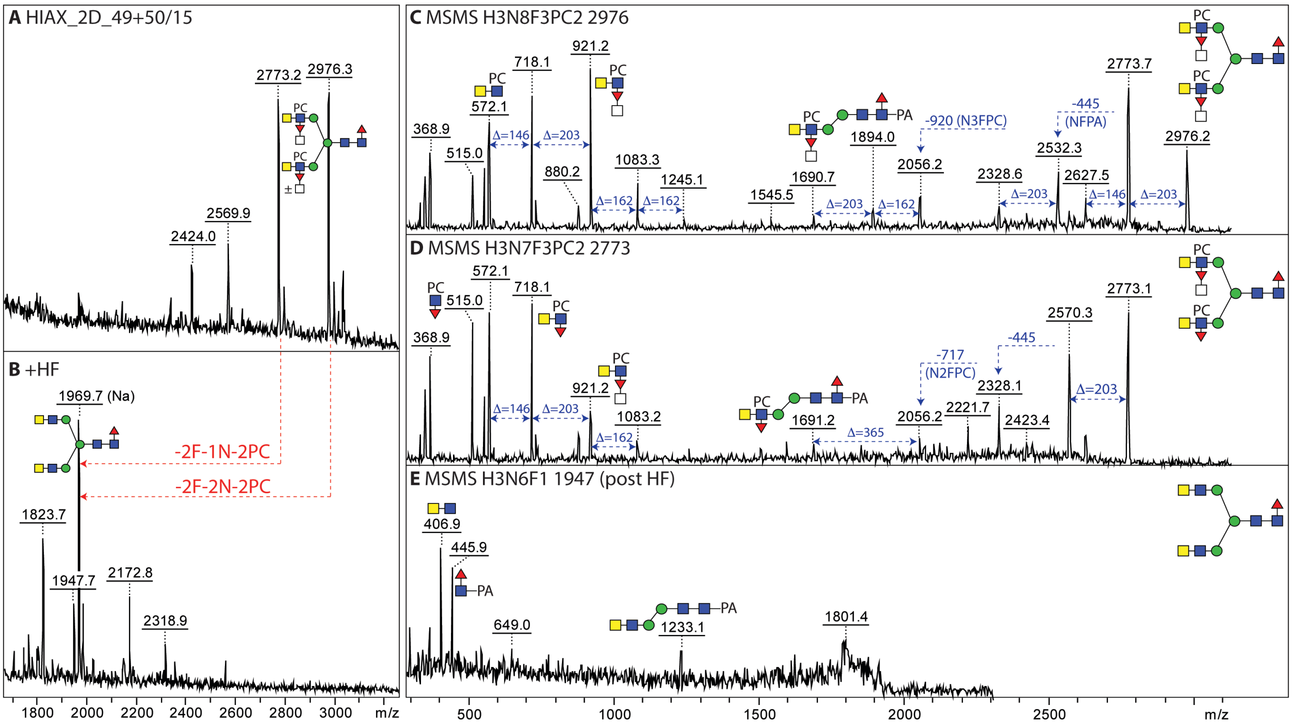

Supplementary Figure 21. N-glycans with phosphorylcholine modifications of mannose and fucosylated LacdiNAc motifs. MALDI-TOF MS/MS of selected N-glycans in different 2D-HPLC fractions (see ***Figure 3 and Supplementary Figure 8***) with multiple phosphorylcholine modifications. (A-G) While the typical PC-modified fucosylated LacdiNAc motif (B2; *m/z* 718), also in substituted form (*m/z* 921), shows the occurrence of antennal phosphorylcholine, putative Y fragments at *m/z* 1154 and 1300 are compatible with a PC-modification of a core α-mannose residue. Furthermore, B fragments at *m/z* 1045 or 1248 as well as *m/z* 328 are also indicative of PCMan motifs; the occurrence of two phosphorylcholine residues on juxtaposed residues results in the formation of ions 59 Da smaller than the true B ion (e.g., fragment ions at *m/z* 986 and 1189); the grey boxes highlight the B3 ions relevant to proposing the isomeric structure (one or two PC residues on one arm). The *m/z* 2938 glycan (panel G) is isobaric to the triantennary pentafucosylated glycan shown in ***Supplementary Figure 10I*** and further isomers of *m/z* 2423, 2569 and 2773 (A-C and F) are respectively shown in ***Supplementary Figure 12 A-F, Figure 4 H-J*** and ***Supplementary Figures 16D-F and 20D.*** The spectra in panels E-G are also shown in ***Figure 5*** of the main text.

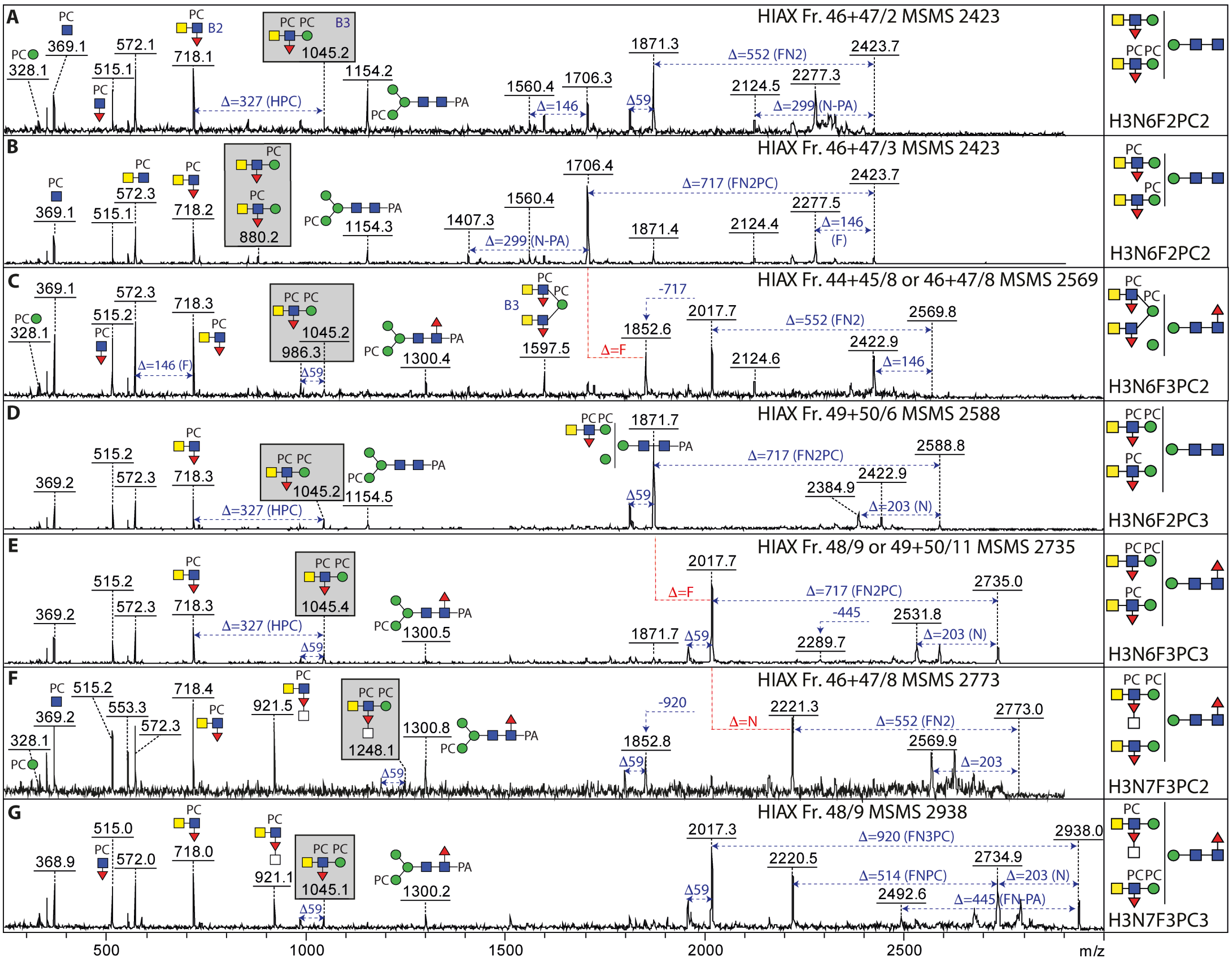

Supplementary Figure 22. Phylogeny of N-glycan branching β1,2/-4/-6-*N*-acetylglucosaminyltransferases in nematodes. As described in the relevant methods section, human and *C. elegans* MGAT1, MGAT2, MGAT3 (human only; Hs), MGAT4 (human only) and MGAT5 sequences were used to search for nematode homologues. For some genes from some species, multiple entries were found; some clades of related sequences are collapsed. The resulting phylogenetic search and tree show that *T. suis* possesses MGAT1, MGAT2 and MGAT4 homologues (Ts MGAT1, Ts MGAT2 and Ts MGAT4), but no MGAT5 homologue could be detected, which contrasts with *C. elegans* which has three MGAT1 (GLY-12, -13 and -14; Ce), one MGAT2 (GLY-20) and one MGAT5 homologues (GLY-2), but no MGAT4; like many other nematodes, the filarial species *Brugia malayi* has at least one homologue each of MGAT1, MGAT2, MGAT4 and MGAT5 (annotated as Bm MGAT1, etc.). No bisecting GlcNAc has been detected in nematodes to date (only bisecting galactose in *C. elegans*), which correlates with a lack of obvious MGAT3 homologues. *C. elegans* has only maximally tri-antennary N-glycans, whereas species such as *Dirofilaria immitis* and *Trichinella spiralis* have 'standard' tetra-antennary N-glycans. Due to the unusual elution properties of the *T. suis* tetra-antennary N-glycans (see ***Supplementary Figures 7 and 13***), a potential explanation is that its MGAT4 can modify both core α-mannose residues with a β1,4-GlcNAc and not just the 'lower arm' α1,3-mannose and so replaces the activity of the absent β1,6-specific MGAT5; however, the predictions of numbers of homologues and their activities requires experimental verification. The Q09327 MGAT3_HUMAN sequence was used as an out group. Bootstrap values are shown under branches. For a full-sized version of the figure, refer to the separate pdf file.

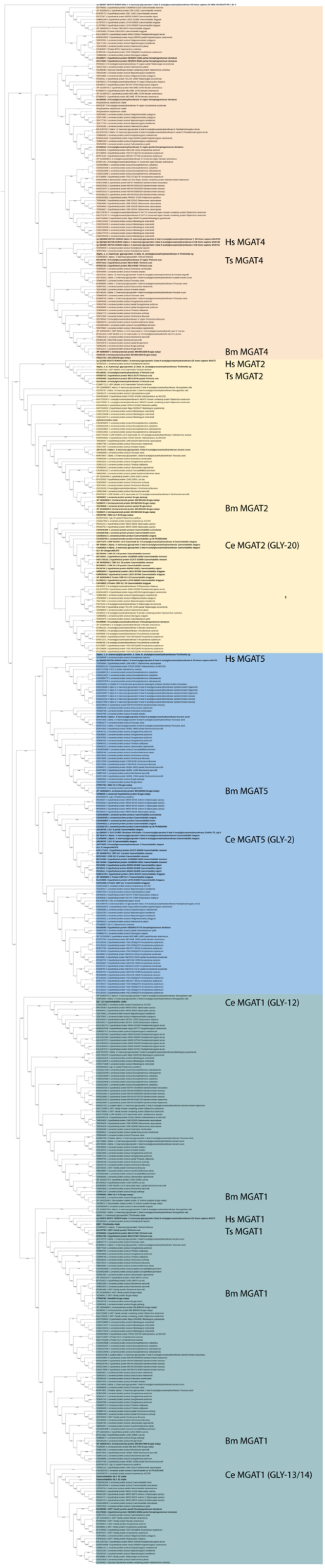

Supplementary Table 1: List of theoretical masses for detected *T. suis* N-glycan structures. Abbreviated compositions of the form $Hex_{3-5}HexNAc_{2-10}Fuc_{0-5}P_{0-1}PC_{0-9}$ and $Hex_{1-10}HexNAc_{2}Fuc_{0-1}$. *m/z* as $[M+H]^+$ for N-glycans labelled with 2-aminopyridine (PA) or phosphorylcholine-modified N-glycans labelled with FMAPA; *m/z* as $[M+Na]^+$ for free N-glycans, N-glycans labelled with 2-aminopyridine (PA) or with FMAPA are given. The numbers of detected potential isomers for the PA-labelled N-glycans and the presence or absence of FMAPA-labelled N-glycans in HPLC fractions are also indicated. Isobaric PC/Fuc- or Fuc-containing compositions are highlighted in blue or red.

Composition	Free [M+Na]+	PA [M+H]+	PA [M+Na]+	FMAPA [M+H]+	FMAPA [M+Na]+	HPLC PA	HPLC FMAPA
H1N2F	755,27	811,34	833,32		1063,43	1	nd
H2N2	771,27	827,34	849,32		1079,43	2	✔
H2N2F	917,32	973,39	995,37		1225,48	2	✔
H3N2	933,32	989,39	1011,37		1241,48	1	✔
H2N3	974,34	1030,41	1052,39		1282,5	1	✔
H3N2F	1079,38	1135,45	1157,43		1387,54	1	✔
H4N2	1095,37	1151,44	1173,42		1403,53	1	✔
H3N3	1136,4	1192,47	1214,45		1444,56	2	✔
H3N2F2	1225,43	1281,5	1303,48		1533,59	1	✔
H4N2F	1241,43	1297,5	1319,48		1549,59	1	✔
H5N2	1257,42	1313,49	1335,47		1565,58	2	✔
H3N3F	1282,46	1338,53	1360,51		1590,62	2	✔
H4N3	1298,38	1354,45	1376,43		1606,54	1	nd
H3N4	1339,48	1395,55	1417,53		1647,64	4	✔
H6N2	1419,48	1475,55	1497,53		1727,64	3	✔
H3N3F2	1428,51	1484,58	1506,56		1736,67	1	✔
H4N3F	1444,51	1500,58	1522,56		1752,67	1	nd
H5N3	1460,5	1516,57	1538,55		1768,66	1	nd
H3N4F	1485,53	1541,6	1563,58		1793,69	6	✔
H4N4	1501,53	1557,6	1579,58		1809,69	1	nd
H3N4PC	1504,53	1560,6	1582,58	1790,71	1812,69	2	✔
H3N5	1542,56	1598,63	1620,61		1850,72	3	nd
H3N4FP	1565,5	1621,57	1643,55	1851,68	1873,66	1	nd
H7N2	1581,53	1637,6	1659,58		1889,69	3	✔
H3N4F2	1631,59	1687,66	1709,64		1939,75	5	✔
H4N4F	1647,59	1703,66	1725,64		1955,75	1	nd
H3N4FPC	1650,59	1706,66	1728,64	1936,77	1958,75	3	✔
H4N4PC	1666,59	1722,66	1744,64	1952,77	1974,75	1	nd
H3N5F	1688,61	1744,68	1766,66		1996,77	4	✔
H3N5PC	1707,61	1763,68	1785,66	1993,79	2015,77	1	nd
H8N2	1743,58	1799,65	1821,63		2051,74	2	✔
H3N6	1745,64	1801,71	1823,69		2053,8	2	✔
H3N4F2PC	1796,65	1852,72	1874,7	2082,83	2104,81	1	✔
H3N4FPC2	1815,65	1871,72	1893,7	2101,83	2123,81	nd	✔
H3N5F2	1834,67	1890,74	1912,72		2142,83	2	✔
H3N5FPC	1853,67	1909,74	1931,72	2139,85	2161,83	1	✔
H3N6F	1891,69	1947,76	1969,74		2199,85	2	✔
H9N2	1905,64	1961,71	1983,69		2213,8	1	✔
H3N6PC	1910,69	1966,76	1988,74	2196,87	2218,85	nd	✔

H3N7	1948,72	2004,79	2026,77		2256,88	2	✓
H3N4F2PC2	1961,7	2017,77	2039,75	2247,88	2269,86	1	✓
H3N6PC-P	1990,66	2046,73	2068,71	2276,84	2298,82	2	nd
H3N5F2PC	1999,73	2055,8	2077,78	2285,91	2307,89	2	✓
H3N5FPC2	2018,73	2074,8	2096,78	2304,91	2326,89	1	✓
H3N6F2	2037,75	2093,82	2115,8		2345,91	1	✓
H3N6FPC	2056,75	2112,82	2134,8	2342,93	2364,91	2	✓
H10N2	2067,69	2123,76	2145,74		2375,85	1	✓
H3N6PC2	2075,75	2131,82	2153,8	2361,93	2383,91	1	✓
H3N7F	2094,77	2150,84	2172,82		2402,93	1	✓
H3N7PC	2113,89	2169,96	2191,94	2400,07	2422,05	nd	✓
H3N6FPCP	2136,72	2192,79	2214,77	2422,90	2444,88	2	nd
H5N4FPC2	2139,93	2196,00	2217,98	2426,11	2448,09	1	nd
H3N8	2151,79	2207,86	2229,84		2459,95	nd	✓
H3N5F2PC2	2164,78	2220,85	2242,83	2450,96	2472,94	1	✓
H3N6F3	2183,81	2239,88	2261,86		2491,97	1	✓
H3N6F2PC	2202,81	2258,88	2280,86	2488,99	2510,97	2	✓
H3N6FPC2	2221,8	2277,87	2299,85	2507,98	2529,96	3	✓
H3N7PC2	2278,83	2334,9	2356,88	2565,01	2586,99	1	✓
H3N8F	2297,85	2353,92	2375,90		2606,01	nd	✓
H3N5F3PC2	2310,96	2367,03	2389,01	2597,14	2619,12	1	nd
H3N8PC	2316,85	2372,92	2394,9	2603,03	2625,01	nd	✓
H3N6F3PC	2348,86	2404,93	2426,91	2635,04	2657,02	1	✓
H3N6F2PC2	2367,86	2423,93	2445,91	2654,04	2676,02	6	✓
H3N6FPC3	2386,86	2442,93	2464,91	2673,04	2695,02	1	nd
H3N7F3	2386,89	2442,96	2464,94		2695,05	1	nd
H3N7FPC2	2424,88	2480,95	2502,93	2711,06	2733,04	2	nd
H3N8FPC	2462,91	2518,98	2540,96	2749,09	2771,07	1	✓
H3N8PC2	2481,91	2537,98	2559,96	2768,09	2790,07	4	✓
H3N6F3PC2	2513,92	2569,99	2591,97	2800,10	2822,08	6	✓
H3N9PC	2520,05	2576,12	2598,1	2806,23	2828,21	2	nd
H3N6F2PC3	2532,92	2588,99	2610,97	2819,10	2841,08	3	✓
H3N7F3PC	2552,02	2608,09	2630,07	2838,20	2860,18	1	nd
H3N10	2557,95	2614,02	2636,00		2866,11	1	✓
H3N7F2PC2	2570,94	2627,01	2648,99	2857,12	2879,1	3	nd
H3N8F3	2589,97	2646,04	2668,02		2898,13	1	nd
H3N8F2PC	2609,05	2665,12	2687,1	2895,23	2917,21	1	nd
H3N8FPC2	2627,96	2684,03	2706,01	2914,14	2936,12	5	✓
H3N8PC3	2646,96	2703,03	2725,01	2933,14	2955,12	nd	✓
H3N9FPC	2666,15	2722,22	2744,2	2952,33	2974,31	2	nd
H3N6F3PC3	2678,98	2735,05	2757,03	2965,16	2987,14	3	✓
H3N9PC2	2684,98	2741,05	2763,03	2971,16	2993,14	3	nd
H3N10F	2704,01	2760,08	2782,06		3012,17	1	nd
H3N7F3PC2	2717,0	2773,07	2795,05	3003,16	3025,16	5	✓
H3N10PC	2723,01	2779,08	2801,06	3009,19	3031,17	1	nd
H3N7F2PC3	2736,0	2792,08	2814,05	3022,18	3044,16	1	
H3N8F4	2736,03	2792,1	2814,08		3044,19	3	nd
H3N8F3PC	2755,02	2811,09	2833,07	3041,20	3063,18	1	nd

H3N8FPC3	2793,02	2849,09	2871,07	3079,20	3101,18	1	✓
H3N9F3	2793,05	2849,12	2871,1		3101,21	3	nd
H3N9FPC2	2831,04	2887,11	2909,09	3117,22	3139,2	1	nd
H3N6F3PC4	2844,03	2900,1	2922,08	3130,21	3152,19	2	✓
H3N9PC3	2850,04	2906,11	2928,09	3136,22	3158,2	1	✓
H3N10FPC	2869,07	2925,14	2947,12	3155,25	3177,23	1	nd
H3N7F3PC3	2882,06	2938,13	2960,11	3168,24	3190,22	1	✓
H3N8F5	2882,06	2938,13	2960,11		3190,22	1	nd
H3N8F4PC	2901,08	2957,15	2979,13	3187,26	3209,24	2	✓
H3N8F3PC2	2920,08	2976,15	2998,13	3206,26	3228,24	4	✓
H3N8F2PC3	2939,08	2995,15	3017,13	3225,26	3247,24	2	✓
H3N9F4	2939,11	2995,18	3017,16		3247,27	3	nd
H3N10PC2P	2968,03	3024,1	3046,08	3254,21	3276,19	2	nd
H3N8F3PC2P	3000,05	3056,12	3078,1	3286,23	3308,21	1	nd
H3N10FPC2	3034,12	3090,19	3112,17	3320,30	3342,28	1	nd
H3N10PC3	3053,12	3109,19	3131,17	3339,30	3361,28	1	✓
H3N8F4PC2	3066,14	3122,21	3144,19	3352,32	3374,3	1	nd
H3N8F3PC3	3085,13	3141,2	3163,18	3371,31	3393,29	2	nd
H3N10PC3P	3133,09	3189,16	3211,14	3419,27	3441,25	1	nd
H3N10F4	3142,18	3198,25	3220,23		3450,34	2	nd
H3N10FPC3	3199,18	3255,25	3277,23	3485,36	3507,34	1	✓
H3N10PC4	3218,18	3274,25	3296,23	3504,36	3526,34	1	✓
H3N8F4PC3	3231,19	3287,26	3309,24	3517,37	3539,35	2	nd
H3N8F3PC4	3250,19	3306,26	3328,24	3536,37	3558,35	1	nd
H3N10FPC3P	3279,14	3335,21	3357,19	3565,32	3587,3	1	nd
H3N10F5	3288,24	3344,31	3366,29		3596,4	1	nd
H3N10F4PC	3307,24	3363,31	3385,29	3593,42	3615,4	2	nd
H3N10F2PC3	3345,23	3401,30	3423,29	3631,39	3653,39	1	✓
H3N11F4	3345,26	3401,33	3423,31		3653,42	1	nd
H3N10FPC4	3364,23	3420,3	3442,28	3650,41	3672,39	1	✓
H3N9F4PC3	3434,27	3490,34	3512,32	3720,45	3742,43	1	nd
H3N10F5PC	3453,27	3509,34	3531,32	3739,45	3761,43	1	nd
H3N10F2PC4	3510,28	3566,35	3588,33	3796,44	3818,40	1	✓
H3N8F4PC5	3561,3	3617,37	3639,35	3847,48	3869,46	1	nd
H3N9F4PC4	3599,33	3655,4	3677,38	3885,51	3907,49	1	✓
H3N10F4PC3	3637,35	3693,42	3715,40	3923,53	3945,51	1	nd
H3N10F3PC4	3656,35	3712,42	3734,4	3942,53	3964,51	1	nd
H3N9F4PC5	3764,38	3820,45	3842,43	4050,56	4072,54	1	✓
H3N9F4PC6	3929,44	3985,51	4007,49	4215,62	4237,6	1	✓
H3N10F5PC4	3948,46	4004,53	4026,51	4234,64	4256,62	1	✓
H3N9F4PC7	4094,49	4150,56	4172,54	4380,67	4402,65	1	✓
H3N10F5PC6	4278,58	4334,65	4356,63	4564,76	4586,74	1	✓
H3N10F5PC7	4443,63	4499,7	4521,68	4729,81	4751,79	1	✓
H3N10F5PC8	4608,69	4664,76	4686,74	4894,87	4916,85	1	✓
H3N10F5PC9	4773,74	4829,81	4851,79	5059,92	5081,90	nd	✓

Supplementary Table 2: List of proposed *T. suis* pyridylaminated N-glycan structures. Abbreviated compositions of the form $Hex_{3\text{-}5}HexNAc_{2\text{-}10}Fuc_{0\text{-}5}P_{0\text{-}1}PC_{0\text{-}8}$ and $Hex_{1\text{-}10}HexNAc_{2}Fuc_{0\text{-}1}$, Glytoucan accessions (in blue; for defined structures up to 2000 Da), *m/z* as $[M+H]^+$, SNFG-style structure, HPLC fraction (either HIAX or, in brackets, HIAX/RP-amide fraction for the second HIAX run) and figure in main text or supplement are given. Structures with a bracket indicate that the exact antennal configuration is not fixed; the HexNAc-substitution of antennal fucose is undefined.

Composition	PA-[M+H]$^+$	Structure	Fraction	Figure
H1N2F1<br>G32152BH	811.34	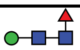	6	
H2N2<br>G81315DD	827.34	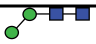	8	
H2N2<br>G22573RC	827.34	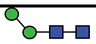	9	
H2N2F<br>G42466VF	973.39	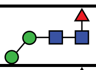	8	
H2N2F<br>G00395TQ	973.39	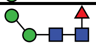	10	
H3N2<br>G22768VO	989.39	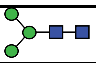	11	
H3N2F<br>G45995IV	1135.45	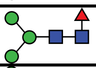	12	
H4N2<br>G09724ZC	1151.44	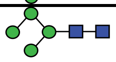	15<br>(9-11/11)	
H3N3<br>G70073SG	1192.47	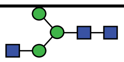	14<br>(9-11/09)	
H3N3<br>G06920GM	1192.47	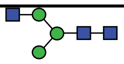	16<br>(9-11/15)	
H3N2F2<br>G77479VH	1281.50	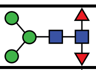	13	
H4N2F<br>G48823SZ	1297.50	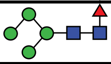	16<br>(9-11/15)	
H5N2<br>G07617FP	1313.49	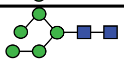	19<br>(12+13/6)	
H5N2<br>G55220VL	1313.49	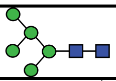	21<br>(12+13/07)	
H3N3F<br>G14576KZ	1338.53	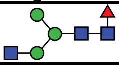	15<br>(9-11/14)	
H3N3F<br>G69987TD	1338.53	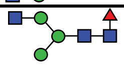	16<br>(9-11/19)	
H4N3<br>G53168IY	1354.45	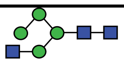	18	
H3N4<br>G20956ZK	1395.55	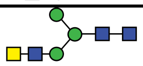	19<br>(12+13/05)	
H3N4<br>G89096IL	1395.55	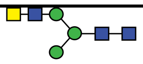	20<br>(12+13/11)	
H3N4<br>G39213VZ	1395.55	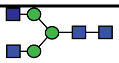	18<br>(12+13/08)	
H3N4<br>G18119VX	1395.55	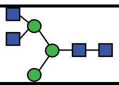	17	
H6N2<br>G61846BY	1475.55	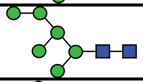	24/25<br>(14-16/07)	
H6N2<br>G80966KZ	1475.55	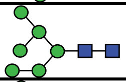	24/25<br>(14-16/08)	
H6N2<br>G34499SX	1475.55	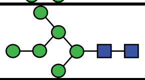	24/25<br>(14-16/12)	
H3N3F2<br>G41720HN	1484.58	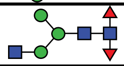	16<br>(9-11/8)	

H3N3F2<br>G10425SO	1484.58	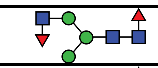	18	
H4N3F<br>G58969RU	1500.58	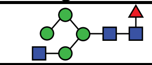	19<br>(12+13/09)	
H5N3<br>G08520NM	1516.57	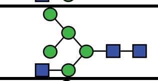	23<br>(14-16/09)	
H3N4F<br>G80019MD	1541.60	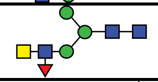	(14-16/06)	
H3N4F<br>G20956ZK	1541.60	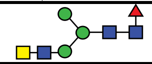	19<br>(12+13/10)	
H3N4F<br>G63770IR	1541.60	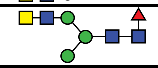	21<br>(12+13/15)	Fig. S9D
H3N4F<br>G80858MF	1541.60	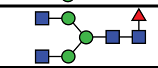	20<br>(12+13/11)	Fig. S9D
H3N4F<br>G92571WK	1541.60	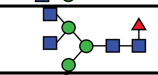	19<br>(12+13/17)	
H4N4<br>G59231NT	1557.60	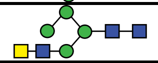	(14-16/10)	
H3N4PC<br>G16427JQ	1560.60	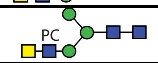 PC	27	
H3N4PC<br>G37840NY	1560.60	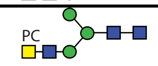 PC	29<br>(17-20/10)	
H3N4PC<br>G87872NF	1560.60	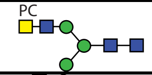 PC	29<br>(17-20/17)	
H3N5<br>G67045LB	1598.63	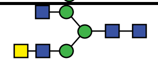	(14-16/13)	
H3N5<br>G19868AX	1598.63	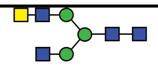	23<br>(14-16/14)	
H3N5	1598.63	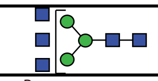	22<br>(14-16/15)	
H3N4FP	1621.57	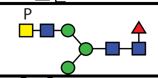 P	35, 36	Fig. S11
H7N2<br>G83161QT	1637.60	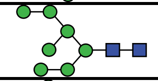	28<br>(17-20/06)	
H7N2<br>G68668TB	1637.60	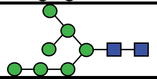	29<br>(17-20/08)	
H7N2<br>G63337SS	1637.60	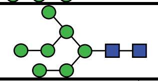	28/29<br>(17-20/11)	
H3N4F2<br>G84692YW	1687.66	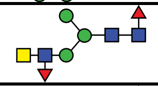	25<br>(14-16/11)	Fig. 4B<br>Fig. S9B
H3N4F2<br>G47253EY	1687.66	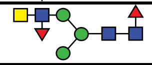	26<br>(14-16/16)	Fig. S9B
H3N4F2<br>G69912LP	1687.66	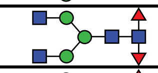	20<br>(12+13/07)	Fig. 4A
H4N4F<br>G45048TZ	1703.66	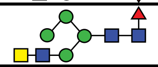	(14-16/14)	
H3N4FPC<br>G04635TH	1706.66	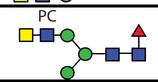 PC	27	Fig. S9C
H3N4FPC<br>G16427JQ	1706.66	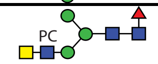 PC	28	
H3N4FPC<br>G67053ZX	1706.66	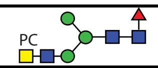 PC	29<br>(17-20/14)	
H3N4FPC<br>G03090TG	1706.66	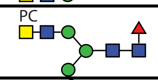 PC	30<br>(17-20/21)	Fig. S9C
H3N4FPC<br>G90339LD	1706.66	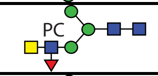 PC	(17-20/06)	Fig. S9C
H4N4PC	1722.66	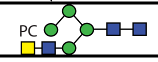 PC	32	

H3N5F<br>G88461FF	1744.68	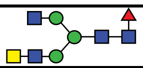	23<br>(14-16/17)	Fig. S9D
H3N5F<br>G82312JY	1744.68	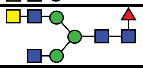	24<br>(14-16/18)	
H3N5F<br>G61207RZ	1744.68	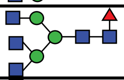	(14-16/19)	Fig. S9D
H3N5F<br>G80630NF	1744.68	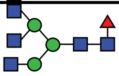	(14-16/24)	
H3N5PC	1763.68	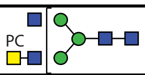	32	
H8N2<br>G40702WU	1799.65	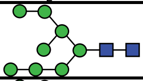	32	
H8N2<br>G89864BN	1799.65	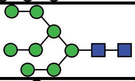	33	
H8N2<br>G91704UR	1799.65	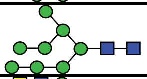	34	
H3N6<br>G09482NW	1801.71	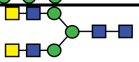	28<br>(17-20/16)	Fig. 4C
H3N6	1801.71	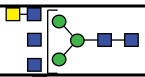	27<br>(14-16/22)	
H3N6<br>G90223GH	1801.71	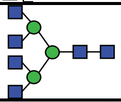	25<br>(14-16/23)	Fig. 4D<br>Fig. S10A
H3N4F2PC<br>G13755PW	1852.72	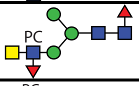	31	Fig. S11A<br>Fig. S15F
H3N4F2PC<br>G21650ZZ	1852.72	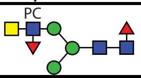	(17-20/16)	
H3N4F2PC<br>G23967QF	1852.72	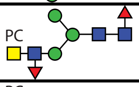	35	
H3N4F2PC<br>G64990BS	1852.72	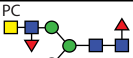	36	Fig. S11E
H3N5F2<br>G30205VB	1890.74	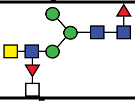	26<br>(17-20/12)	Fig. 5A<br>Fig. S19A
H3N5F2	1890.74	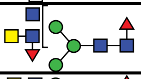	27<br>(17-20/17)	Fig. S10F
H3N5F2<br>G55860VC	1890.74	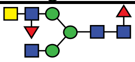	29<br>(17-20/15)	Fig. S10E
H3N5FPC	1909.74	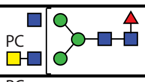	32	
H3N5FPC	1909.74	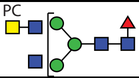	34	
H3N6F<br>G85542KD	1947.76	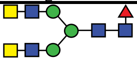	(17-20/22)	
H3N6F	1947.76	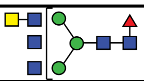	27	
H3N6F<br>G59866SG	1947.76	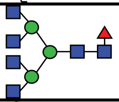	26<br>(17-20/26)	
H9N2<br>G60230HH	1961.71	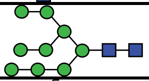	37	
H3N7	2004.79	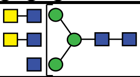	(17-20/19)	Fig. S10B
H3N7	2004.79	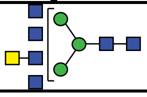	(17-20/24)	Fig. S10C

H3N4F2PC2	2017.77	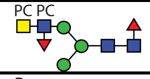	40	
H3N6PCP	2046.73	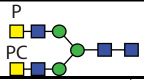	44+45<br>46+47	Fig. S17F
H3N5F2PC	2055.80	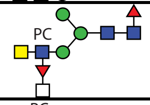	31, 32	Fig. 5B<br>Fig. S15E<br>Fig. S19B
H3N5F2PC	2055.80	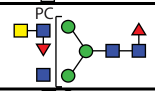	34	
H3N5F2PC	2055.80	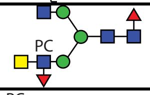	36	
H3N5F2PC	2055.80	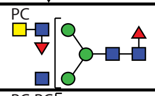	38	Fig. S15A
H3N5FPC2	2074.80	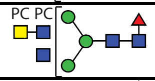	39	
H3N6F2	2093.82	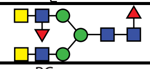	39	
H3N6FPC	2112.82	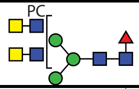	34	
H3N6FPC	2112.82	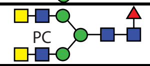	35	
H10N2<br>G19958IL	2123.76	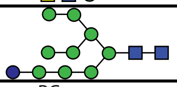	40	
H3N6PC2	2131.82	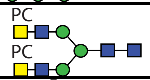	44+45	Fig. S17G
H3N7F	2150.84	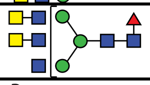	31	
H3N6FPCP	2192.79	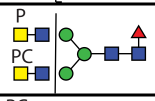	44+45	Fig. S17A
H3N6FPCP	2192.79	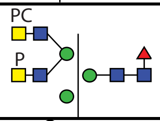	46+47	Fig. S17D
H5N4FPC2	2196.00	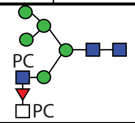	46+47	Fig. S19G
H3N5F2PC2	2220.85	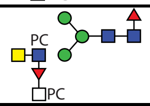	40	Fig. 5C<br>Fig. S19C
H3N6F3	2239.88	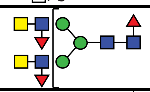	39	Fig. S10G
H3N6F2PC	2258.88	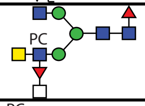	36	
H3N6F2PC	2258.88	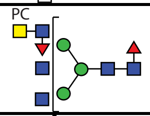	38	
H3N6F2PC	2258.88	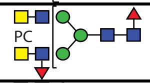	42	
H3N6F2PC	2258.88	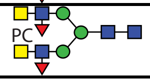	46+47	

H3N6FPC2	2277.87	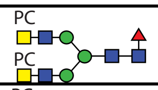	44+45	Fig. 4E<br>Fig. S17C
H3N6FPC2	2277.87	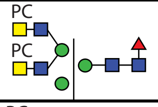	44+45	Fig. S18A
H3N6FPC2	2277.87	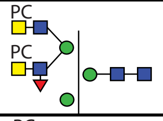	46+47	Fig. S17E
H3N7PC2	2334.90	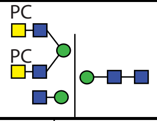	46+47	
H3N6F3PC	2404.93	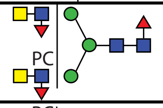	43, 44+45	Fig. 5E<br>Fig. S19E
H3N6F2PC2	2423.93	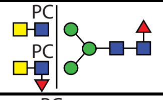	44+45	
H3N6F2PC2	2423.93	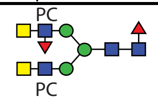	44+45	Fig. S12E
H3N6F2PC2	2423.93	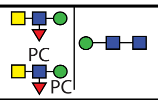	46+47	Fig. S21A
H3N6F2PC2	2423.93	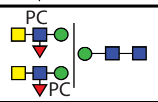	46+47	Fig. S21B
H3N6F2PC2	2423.93	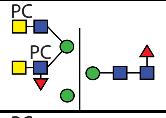	46+47	Fig. S12D
H3N6F2PC2	2423.93	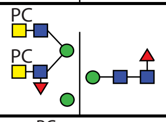	46+47	Fig. S12F
H3N6F2PC2	2423.93	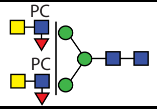	48	Fig. S12B
H3N6F2PC2	2423.93	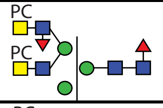	48	
H3N6F2PC2	2423.93	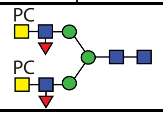	49+50	Fig. S12A
H3N6F2PC2	2423.93	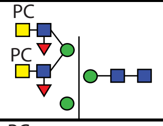	49+50	Fig. S12C
H3N6FPC3	2442.93	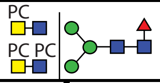	48<br>49+50	Fig. 4F<br>Fig. S12G
H3N7F3	2442.96	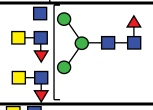	39	
H3N7FPC2	2480.95	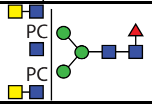	46+47	

H3N7FPC2	2480.95	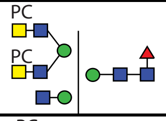	46+47	
H3N8FPC	2518.98	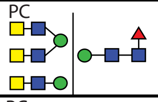	44+45	
H3N8PC2	2537.98	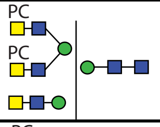	48<br>49+50	
H3N8PC2	2537.98	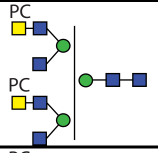	48	
H3N8PC2	2537.98	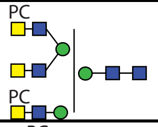	49+50	
H3N6F3PC2	2569.99	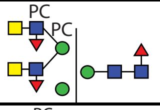	44+45<br>46+47	Fig. S21C
H3N6F3PC2	2569.99	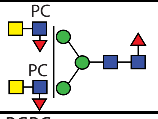	48<br>49+50	Fig. 4G<br>Fig. S12H
H3N6F3PC2	2569.99	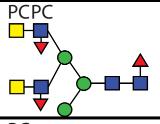	49+50	Fig. 4H<br>Fig. S12I
H3N6F3PC2	2569.99	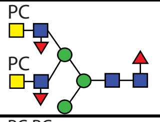	49+50	Fig. 4I<br>Fig. S12J
H3N6F3PC2	2569.99	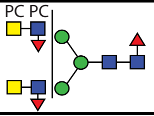	51+52	
H3N9PC	2576.12	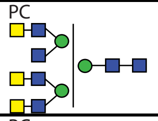	44+45	
H3N9PC	2576.12	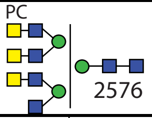	44+45	
H3N6F2PC3	2588.99	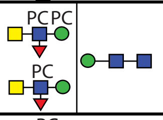	49+50	Fig. S21D
H3N6F2PC3	2588.99	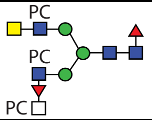	51+52	
H3N6F2PC3	2588.99	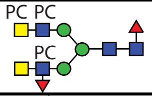	51+52	
H3N7F3PC	2608.09	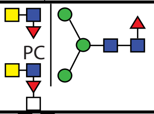	44+45	Fig. 5D<br>Fig. S19D
H3N10<br>G53059NZ	2614.02	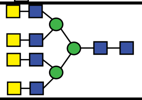	41	

H3N7F2PC2	2627.01	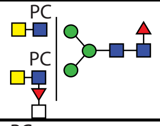	44+45	
H3N7F2PC2	2627.01	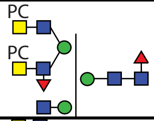	49+50	
H3N7F2PC2	2627.01	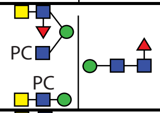	48	
H3N8F3	2646.04	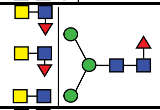	44+45	
H3N8F2PC	2665.12	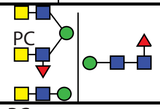	46+47	
H3N8FPC2	2684.03	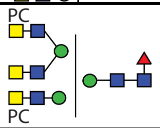	46+47	Fig. S16A
H3N8FPC2	2684.03	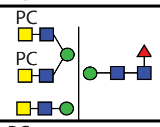	48<br>49+50	Fig. S16B
H3N8FPC2	2684.03	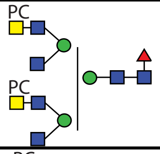	49+50	Fig. S16C
H3N9FPC	2722.22	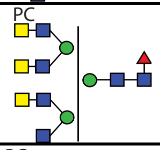	46+47	
H3N9FPC	2722.22	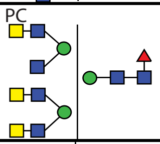	46+47	
H3N6F3PC3	2735.05	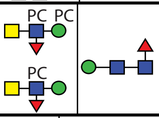	48<br>49+50	Fig. 5G<br>Fig. S21E
H3N6F3PC3	2735.05	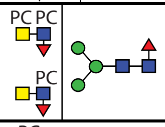	56+57	
H3N9PC2	2741.05	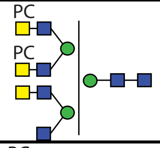	49+50<br>51+52	
H3N9PC2	2741.05	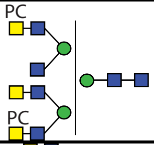	49+50<br>51+52	
H3N10F<br>G51374ZY	2760.08	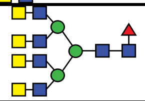	43	

H3N7F3PC2	2773.07	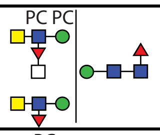	46+47<br>51+52	Fig. 5H<br>Fig. S21F
H3N7F3PC2	2773.07	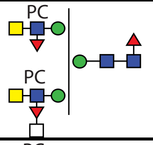	48<br>49+50	Fig. S16E<br>Fig. S20D
H3N7F3PC2	2773.07	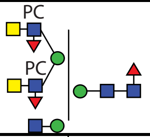	46+47	Fig. S16F
H3N7F3PC2	2773.07	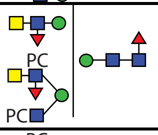	51+52	Fig. S16D
H3N7F3PC2	2773.07	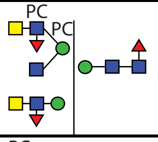	51+52	
H3N10PC	2779.08	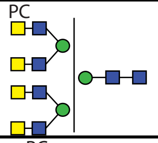	48	
H3N7F2PC3	2792.08	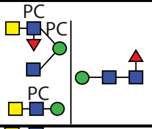	51+52	
H3N8F4	2792.10	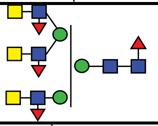	48	Fig. S10H
H3N8F4	2792.10	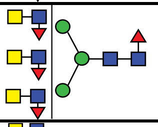	49+50	
H3N8F3PC	2811.09	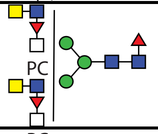	44+45	
H3N8FPC3	2849.09	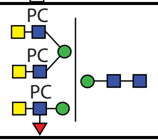	51+52	
H3N8FPC3	2849.09	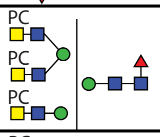	53-55	Fig. S18B
H3N8FPC3	2849.09	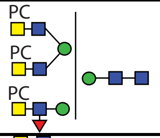	56+57	
H3N9F3	2849.12	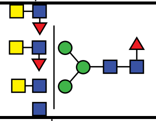	44+45	
H3N9F3	2849.12	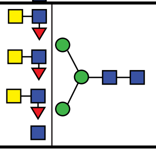	48	

H3N9FPC2	2887.11	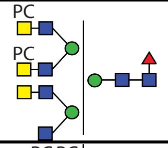	51+52	
H3N6F3PC4	2900.10	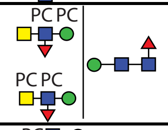	53-55	
H3N6F3PC4	2900.10	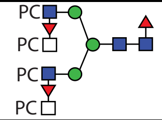	58+59	Fig. S19H
H3N9PC3	2906.11	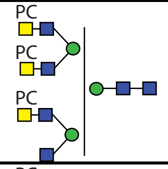	53-55	
H3N10FPC	2925.14	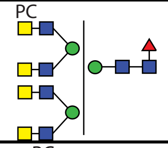	48	
H3N7F3PC3	2938.13	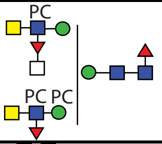	48	Fig. 5I<br>Fig. S21G
H3N8F5	2938.13	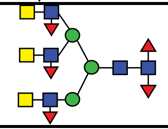	48	Fig. S10I
H3N8F4PC	2957.15	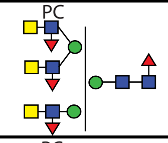	51+52	
H3N8F3PC2	2976.15	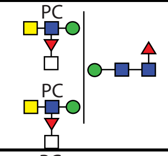	49+50	Fig. S20C
H3N8F3PC2	2976.15	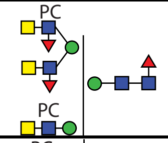	51+52	
H3N8F3PC2	2976.15	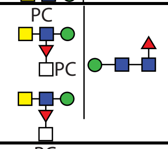	51+52	
H3N8F3PC2	2976.15	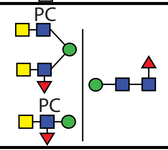	51+52	
H3N8F3PC2	2976.15	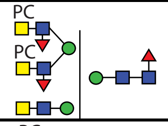	53-55	
H3N8F2PC3	2995.15	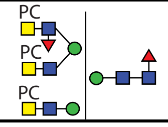	56+57	Fig. S16J

H3N8F2PC3	2995.15	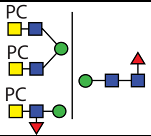	56+57	
H3N9F4	2995.18	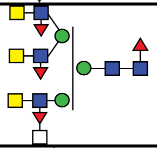	48<br>49+50	Fig. S16G
H3N9F4	2995.18	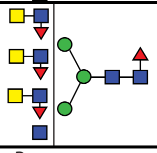	48<br>49+50<br>51+52	Fig. S16I
H3N10PC2P	3024.10	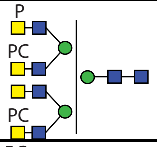	56+57	
H3N10PC2P	3024.10	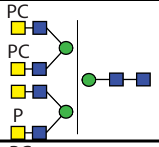	56+57	Fig. S17H
H3N8F3PC2P	3056.12	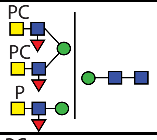	56+57	
H3N10FPC2	3090.19	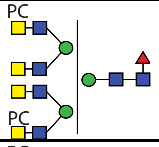	51+52	
H3N10PC3	3109.19	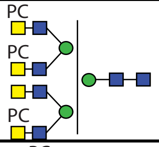	56+57	Fig. S17I
H3N8F4PC2	3122.21	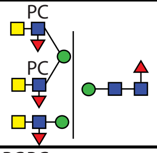	53-55	
H3N8F4PC2	3122.21	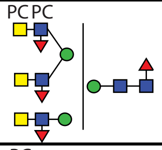	56+57	
H3N8F3PC3	3141.20	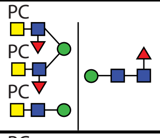	58+59	
H3N8F3PC3	3141.20	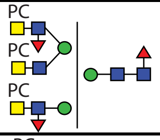	58+59	
H3N10PC3P	3189.16	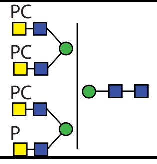	58+59<br>60+61	Fig. S13

H3N10F4	3198.25	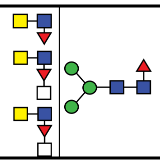	49+50	
H3N10F4	3198.25	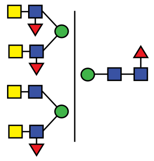	51+52	
H3N10F4	3198.25	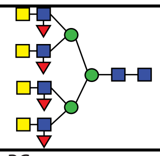	53-55	
H3N10FPC3	3255.25	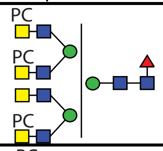	56+57	Fig. S18C
H3N10PC4	3274.25	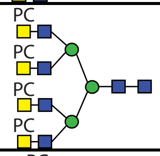	60+61	Fig. S13
H3N8F4PC3	3287.26	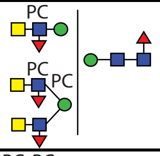	56+57	Fig. S18F
H3N8F4PC3	3287.26	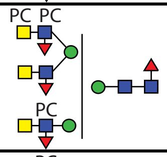	56+57<br>58+59	Fig. S18G
H3N8F3PC4	3306.26	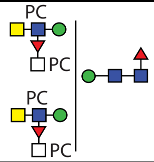	56+57	
H3N10FPC3P	3335.21	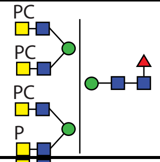	58+59	Fig. S17J
H3N10F5<br>G41784WX	3344.31	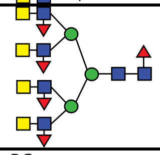	53-55	Fig. S10J
H3N10F4PC	3363.31	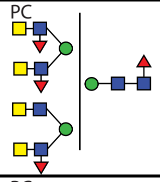	58+59	
H3N10F4PC	3363.31	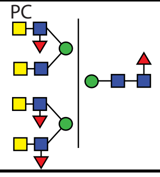	58+59	

H3N10F2PC3	3401.30	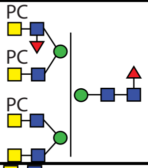	58+59	Fig. S18D
H3N11F4	3401.33	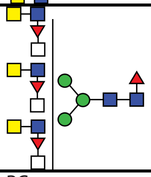	49+50	Fig. S18E
H3N10FPC4	3420.30	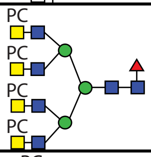	60+61	Fig. S13
H3N9F4PC3	3490.34	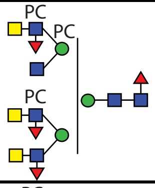	56+57	Fig. S18H
H3N9F4PC3	3490.34	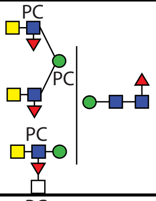	56+57	Fig. S18I
H3N10F5PC	3509.34	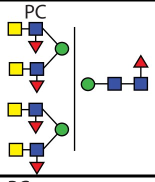	56+57	
H3N10F5PC	3509.34	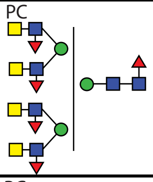	58+59	
H3N10F2PC4	3566,35	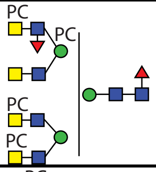	61	
H3N8F4PC5	3617.37	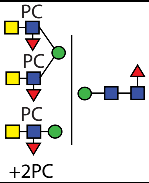	66	
H3N9F4PC4	3655.40	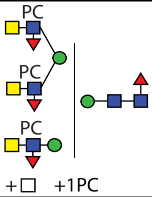	62	

H3N10F4PC3	3693.42	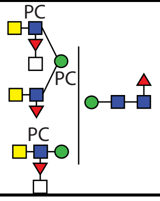	56+57	
H3N10F3PC4	3712.42		65	
H3N9F4PC5	3820.45		66	
H3N9F4PC6	3985.51	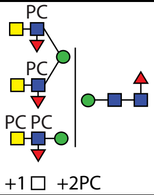	71	
H3N10F5PC4	4004.53		67	
H3N9F4PC7	4150.56		73	
H3N10F5PC6	4334.65		73	
H3N10F5PC7	4499.70	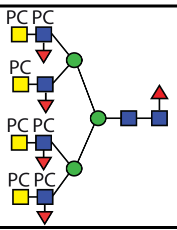	75	Fig. S14
H3N10F5PC8	4664.76		75	
